# Association of Prenatal Exposure to Population-Wide Folic Acid Fortification With Altered Cerebral Cortex Maturation in Youths

**DOI:** 10.1001/jamapsychiatry.2018.1381

**Published:** 2018-07-03

**Authors:** Hamdi Eryilmaz, Kevin F. Dowling, Franklin C. Huntington, Anais Rodriguez-Thompson, Thomas W. Soare, Lauren M. Beard, Hang Lee, Jeffrey C. Blossom, Randy L. Gollub, Ezra Susser, Ruben C. Gur, Monica E. Calkins, Raquel E. Gur, Theodore D. Satterthwaite, Joshua L. Roffman

**Affiliations:** 1 Department of Psychiatry, Massachusetts General Hospital, Harvard Medical School, Charlestown; 2 Penn–Children’s Hospital of Philadelphia Lifespan Brain Institute, Department of Psychiatry, Perelman School of Medicine, University of Pennsylvania, Philadelphia; 3 Massachusetts General Hospital Biostatistics Center, Harvard Medical School, Boston; 4 Center for Geographic Analysis, Harvard University, Cambridge, Massachusetts; 5 Department of Epidemiology, Columbia University, New York, New York; 6 Department of Psychiatry, Columbia University, New York, New York; 7 New York State Psychiatric Institute, New York, New York

## Abstract

**Importance:**

Presently, 81 countries mandate the fortification of grain products with folic acid to lessen the risk of neural tube defects in the developing fetus. Epidemiologic data on severe mental illness suggest potentially broader effects of prenatal folate exposure on postnatal brain development, but this link remains unsubstantiated by biological evidence.

**Objective:**

To evaluate associations among fetal folic acid exposure, cortical maturation, and psychiatric risk in youths.

**Design, Setting, and Participants:**

A retrospective, observational clinical cohort study was conducted at Massachusetts General Hospital (MGH) among 292 youths 8 to 18 years of age born between January 1993 and December 2001 (inclusive of folic acid fortification rollout ±3.5 years) with normative results of clinical magnetic resonance imaging, divided into 3 age-matched groups based on birthdate and related level of prenatal folic acid fortification exposure (none, partial, or full). Magnetic resonance imaging was performed between January 2005 and March 2015. Two independent, observational, community-based cohorts (Philadelphia Neurodevelopmental Cohort [PNC] and National Institutes of Health Magnetic Resonance Imaging Study of Normal Brain Development [NIH]) comprising 1078 youths 8 to 18 years of age born throughout (PNC, 1992-2003) or before (NIH, 1983-1995) the rollout of folic acid fortification were studied for replication, clinical extension, and specificity. Statistical analysis was conducted from 2015 to 2018.

**Exposures:**

United States–mandated grain product fortification with folic acid, introduced in late 1996 and fully in effect by mid-1997.

**Main Outcomes and Measures:**

Differences in cortical thickness among nonexposed, partially exposed, and fully exposed youths (MGH) and underlying associations between age and cortical thickness (all cohorts). Analysis of the PNC cohort also examined the association of age–cortical thickness slopes with the odds of psychotic symptoms.

**Results:**

The MGH cohort (139 girls and 153 boys; mean [SD] age, 13.3 [2.3] years) demonstrated exposure-associated cortical thickness increases in bilateral frontal and temporal regions (9.9% to 11.6%; corrected *P* < .001 to *P* = .03) and emergence of quadratic (delayed) age-associated thinning in temporal and parietal regions (β = –11.1 to –13.9; corrected *P* = .002). The contemporaneous PNC cohort (417 girls and 444 boys; mean [SD] age, 13.5 [2.7] years) also exhibited exposure-associated delays of cortical thinning (β = –1.59 to –1.73; corrected *P* < .001 to *P* = .02), located in similar regions and with similar durations of delay as in the MGH cohort. Flatter thinning profiles in frontal, temporal, and parietal regions were associated with lower odds of psychosis spectrum symptoms in the PNC cohort (odds ratio, 0.37-0.59; corrected *P* < .05). All identified regions displayed earlier thinning in the nonexposed NIH cohort (118 girls and 99 boys; mean [SD] age, 13.3 [2.6] years).

**Conclusions and Relevance:**

The results of this study suggest an association between gestational exposure to fortification of grain products with folic acid and altered cortical development and, in turn, with reduction in the risk of psychosis in youths.

## Key Points

**Question:**

Is increased fetal exposure to folic acid, implemented through population-wide fortification of grain products, associated with clinically meaningful changes in postnatal brain development?

**Findings:**

In a cohort of 292 youths 8 to 18 years of age with normative results of clinical magnetic resonance imaging, delayed age-associated thinning of the cerebral cortex, a pattern suggesting reduced risk for severe mental illness, emerged among individuals who gestated during and after the fortification rollout in the United States (1996-1997). Studies of 2 additional independent US cohorts (N = 1078) confirmed the reliability and temporal specificity of fortification-associated delays of cortical thinning and demonstrated an associated reduction in psychosis risk.

**Meaning:**

Beyond its known association with the prevention of neural tube defects, increased gestational exposure to folic acid through food fortification may protect against psychosis through altered postnatal cortical development.

## Teaser

This cohort study evaluates associations among fetal folic acid exposure through fortification of grain products, cortical maturation, and psychiatric risk in youths.

## Introduction

In March 1996, the US government mandated that all food manufacturers fortify enriched grain products with 140 μg of folic acid per 100 g of food by January 1, 1998.^1^ This intervention was implemented to provide increased fetal exposure to folic acid (a synthetic and more highly bioavailable form of naturally occurring folate) in the first month of gestation, a time critical to neural tube development but before many pregnancies are recognized. The fortification rollout rapidly doubled blood folate levels in women of childbearing age^2^ and substantially diminished neural tube defects in newborns.^3,4^ At present, 81 countries require folic acid fortification of grain products (eFigure 1 in the Supplement).

Folate may play other important roles in the development of the fetal central nervous system, given its contributions to DNA synthesis, DNA and histone methylation, and gene expression. The hypothesis that prenatal exposure to folate may also influence postnatal brain development arises in part from epidemiologic studies that linked starvation during early fetal life with both neural tube defects and schizophrenia,^5^ and is further supported by studies that linked periconceptional folic acid supplements to lower risk of language delay and autism^6,7,8,9^; however, 1 study failed to find such an association.^10^ A critical unanswered question is whether variation in fetal exposure to folate subsequently influences brain development during the formative years preceding late adolescence and early adulthood, a period associated with heightened risk for psychiatric disorders.

The present study used the US rollout of folic acid fortification of grain products to examine the association between increased fetal exposure to folic acid and subsequent cortical development. Our primary measurement was cortical thickness obtained from magnetic resonance imaging (MRI) scans because it provides a clinically relevant developmental marker. Studies of healthy pediatric samples reveal a steady age-associated decrease in thickness across most of the cortical mantle,^11^ a pattern thought to reflect synaptic pruning^12^ and cortical myelination.^13,14^ Whereas the trajectory of thinning is typically linear,^11^ departures from this pattern can have functional consequences; delayed onset of thinning has been associated with higher intelligence,^15^ but accelerated loss of gray matter has been described in patients with schizophrenia^16^ and their unaffected relatives,^17^ as well as in school-aged children with autism.^18^

Using data from normative clinical brain MRI scans accessed from the Massachusetts General Hospital (MGH),^19^ we compared cortical thickness indices within a large cohort of youths born just before, during, or just after the rollout of folic acid fortification and who, therefore, would have received progressively greater exposure to folic acid during gestation. Although little, if any, fortification was in place by September 1996, its rapid deployment ensured that the transition was nearly complete within New England by July 1997.^20^ Therefore, comparison groups were predefined based on date of birth, so that no individuals in the pre-rollout group (born prior to July 1, 1996) were exposed to fortification during any part of gestation, every individual in the post-rollout group (born after June 30, 1998) was exposed during the entire pregnancy, and individuals in the rollout group (born between these dates) were intermediately exposed. We then turned to 2 additional large, US-based pediatric MRI repositories, the Philadelphia Neurodevelopmental Cohort (PNC) and the National Institutes of Health MRI Study of Normal Brain Development (NIH), to test the reliability and specificity of fortification-related associations with cortical development, and the relevance of fortification-associated MRI changes to psychopathologic characteristics.

## Methods

### MGH Cohort

Patients with brain MRI scans were identified through purposeful sampling of the MGH medical record (eFigure 2A in the Supplement). The search returned 3311 radiology reports, based on both general inclusion criteria (8.0-18.0 years of age at time of scan, date of birth between January 1993 and December 2001, and MRI scans occurring between January 2005 and March 2015) and a predetermined algorithm to optimize age matching of groups. After excluding MRI scans with abnormalities that were identified in the corresponding radiology reports (eTable 1 in the Supplement), and then subjecting the remaining scans to stringent quality control procedures blinded to birthdate (eFigure 2B in the Supplement), we arrived at 292 usable, clinically normative scans, comprising 97 pre-rollout (nonexposed), 96 rollout (partially exposed), and 99 post-rollout (fully exposed) unique individuals (Table 1 and the eAppendix in the Supplement). Study procedures were approved by Partners Human Research Committee, which granted a waiver of informed consent, since this retrospective study of the medical record involved only deidentified data.

**Table 1.** Characteristics of Massachusetts General Hospital Cohort Participants

Characteristic	Participants, No. (%)			Statistics	*P* Value
	Nonexposed (n = 97)	Partially Exposed (n = 96)	Fully Exposed (n = 99)		
**Participant-Level Data (RPDR)**					
Age, mean (SD), y	13.3 (2.1)	13.5 (2.8)	12.9 (2.0)	*F* = 1.72	.18
Female sex	49 (50.5)	43 (44.8)	47 (47.5)	χ^2^ = 0.67	.72
Race/ethnicity					
African American	6 (6.2)	3 (3.1)	6 (6.1)	FE = 6.21	.81
Asian	5 (5.2)	2 (2.1)	3 (3.0)		
White	71 (73.2)	69 (71.9)	73 (73.7)		
Hispanic	9 (9.3)	11 (11.5)	7 (7.1)		
Not recorded	2 (2.1)	6 (6.3)	6 (6.1)		
Other	4 (4.1)	5 (5.2)	4 (4.0)		
Insurance					
Private	58 (59.8)	48 (50.0)	67 (67.6)	FE = 6.41	.13
Public	38 (39.2)	46 (47.9)	31 (31.3)		
Other	1 (1.0)	2 (2.1)	1 (1.0)		
Scanner					
1.5-T General Electric	66 (68.0)	41 (42.7)	25 (25.3)	FE = 45.5	<.001
1.5-T Siemens Avanto	6 (6.2)	2 (2.1)	3 (3.0)		
1.5-T Siemens Aera	0	0	1 (1.0)		
3.0-T Siemens Trio	24 (24.7)	50 (52.1)	67 (67.7)		
3.0-T Siemens Skyra	1 (1.0)	3 (3.1)	3 (3.0)		
**Neighborhood Block-Level Data (ACS)**^a^					
Household income, median, $	81 252	79 368	85 361	*F* = 0.59	.59
Unemployment	8.6	9.7	8.8	*F* = 1.28	.28
Highest educational level					
No high school	5.4	5.3	4.1	Group: *F* = 1.02; group × level: *F* = 0.53	.36; .89
Some high school	6.0	6.0	5.5		
High school graduate	26.3	26.9	26.2		
Some college	15.2	14.4	14.2		
Associates degree	7.2	7.7	7.3		
Bachelor’s degree	22.2	21.0	23.6		
Graduate degree	17.8	17.4	19.0		
Vitamin consumption					
Households reporting use (index)	105.6	104.6	107.3	*F* = 0.76	.47
Spending per household, $	76.6	76.1	82.3	*F* = 0.81	.45
**Reason for MRI Scan**					
Attention-deficit/hyperactivity disorder	1 (1.0)	1 (1.0)	0	FE = 1.28	.55
Altered mental status	9 (9.3)	4 (4.2)	9 (9.1)	FE = 2.44	.34
Ataxia	4 (4.1)	5 (5.2)	2 (2.0)	FE = 1.46	.44
Autism	3 (3.1)	9 (9.4)	7 (7.1)	FE = 3.28	.18
Cognitive delay or learning disability	2 (2.1)	5 (5.2)	8 (8.1)	FE = 3.60	.16
Family history of neurologic disorder	0	2 (2.1)	1 (1.0)	FE = 1.86	.44
Focal neurologic finding	8 (8.2)	13 (13.5)	11 (11.1)	χ^2^ = 1.39	.50
Head injury	8 (8.2)	7 (7.3)	9 (9.1)	χ^2^ = 0.21	.96
Non-CNS tumor or surgery	3 (3.1)	3 (3.1)	3 (3.0)	FE = 0.14	>.99
Not given	1 (1.0)	1 (1.0)	0	FE = 1.28	.55
Pituitary or endocrine	1 (1.0)	3 (3.1)	9 (9.1)	FE = 7.48	.02
Psychosis	0	2 (2.1)	0	FE = 2.71	.11
Seizures or epilepsy	21 (21.6)	29 (30.2)	27 (27.3)	χ^2^ = 1.88	.40
Somatic symptoms^b^	48 (49.5)	37 (38.5)	45 (45.5)	χ^2^ = 2.39	.31
Syncope	2 (2.1)	4 (4.2)	2 (2.0)	FE = 1.05	.61
**Previous Medication Use**					
Psychotropic medications					
Anticonvulsants	15 (15.5)	19 (19.8)	19 (19.4)	χ^2^ = 0.74	.70
Antidepressants	11 (11.3)	12 (12.5)	17 (17.2)	χ^2^ = 1.58	.46
Antipsychotics	8 (8.2)	7 (7.3)	7 (7.1)	χ^2^ = 0.11	.96
Benzodiazepines	11 (11.3)	14 (14.6)	20 (20.2)	χ^2^ = 3.03	.23
Stimulants	3 (3.1)	6 (6.3)	11 (11.1)	FE = 4.81	.08
Folic acid or multivitamin	2 (2.1)	2 (2.1)	2 (2.0)	FE = 0.22	>.99

Abbreviations: ACS, 2010 American Community Survey; CNS, central nervous system; FE, Fisher exact test (used in lieu of the χ^2^ test when <5 patients appeared in at least 1 cell); MRI, magnetic resonance imaging; RPDR, Research Patient Data Registry.

^a^ Only percentage data, not numbers of individuals, were available from the ACS.

^b^ Somatic symptoms: nausea, headache, and/or dizziness.

Although rapid implementation of the fortification rollout and use of age-matched comparison groups diminished the risk for temporal confounding, we assessed numerous factors that could potentially influence any between-group differences in cortical thickness. To account for socioeconomic and biological diversity in the sample, we extracted from the electronic medical record demographic and socioeconomic information (age at scan, sex, race/ethnicity, and public vs private insurance); reason for MRI scan; and previous use of psychotropic medications, folic acid, or multivitamins. To provide additional measures of socioeconomic status and vitamin use, we performed geospatial analysis to tag patients’ last known addresses to block-level data from the 2010 American Community Survey.^21^ These data included median household income, household educational attainment, unemployment rate, vitamin intake, and vitamin-related spending (eAppendix in the Supplement).

### PNC Cohort

The PNC participants have been described elsewhere.^22^ In brief, participants included here comprised a subset of 861 individuals, 8.0 to 18.0 years of age, recruited from community health settings in Philadelphia, Pennsylvania. Participants underwent standardized clinical and MRI assessment using a single 3-T magnet. Clinical assessment^23^ characterized participants as either typically developing or exhibiting psychiatric symptoms, categorized as psychosis spectrum, attenuated psychosis, or other types of psychopathologic conditions (eAppendix in the Supplement). All included MRI scans passed stringent quality control as previously described.^24^ The distribution of birthdates among 8- to 18-year-olds in the PNC sample was such that nonexposed (n = 322), partially exposed (n = 189), and fully exposed (n = 350) individuals were well represented (eFigure 3 in the Supplement). Study procedures were approved by the institutional review boards of the Children’s Hospital of Philadelphia and the University of Pennsylvania. Adult participants provided written informed consent. Minors provided assent, and their parent or guardian provided written informed consent.

### NIH Cohort

The NIH participants have been characterized elsewhere.^25^ In brief, healthy youths were recruited across 6 sites nationwide and underwent MRI scans on 1.5-T magnets up to 3 times at various ages. Here, we selected a subsample from this cohort whose MRI scans previously passed stringent image quality control,^11^ and we constrained it to our age interval of interest (8.0-18.0 years). We also excluded participants who might have been exposed to folic acid fortification based on their age at first enrollment. The final sample included 217 individual participants and 383 MRI scans. All procedures were approved by the relevant institutional review board at each of the 6 pediatric study centers, where the MRI scans took place, at a clinical coordinating center at Washington University in St Louis, and at a data coordinating center at the Montreal Neurological Institute, McGill University. Written informed consent was obtained from parents and adult participants, and minors provided assent.

### Statistical Analysis

Primary analyses used general linear models in FreeSurfer, version 5.0 (Martinos Center for Biomedical Imaging). Main analyses in the MGH cohort contrasted mean cortical thickness and age-associated change in thickness (linear and quadratic models) in the fully exposed vs nonexposed groups. Main analyses in the contemporaneous PNC cohort and comparison NIH cohort assessed for significant quadratic associations of age with cortical thickness across each cohort. Nuisance variables, including age, sex, total brain volume (all cohorts), scanner field strength (MGH), and site (NIH), were entered as covariates of no interest. To ensure coverage of the entire cortex, we did not limit the analysis to a priori anatomical regions of interest. Rather, we used 10 000 Monte Carlo simulations to determine whether identified clusters bounded by a vertexwise threshold of *P* < .05 were sufficiently large to survive control for multiple comparisons across the entire surface (clusterwise *P* < .05). For clusters demonstrating significant quadratic associations of age with thickness, the delay in cortical thinning (ie, time until thinning onset) was estimated using least squares analyses (MATLAB, version R2015b; Mathworks Inc). To assess the associations of cortical thinning delay with clinical outcomes in the PNC cohort, multinomial logistic regression examined the association of the local age-thickness slope with adjusted odds for diagnosis of psychosis spectrum, attenuated psychosis, and other types of psychopathologic conditions compared with typically developing participants (SPSS, version 25 [SPSS Inc]; eAppendix in the Supplement).

## Results

### MGH Cohort

Included and excluded patients were comparable across demographic measures (eTable 2 in the Supplement). Among included individuals, exposure groups did not differ significantly by age at MRI scan, sex, scan indication, or insurance status; a slight but nonsignificant increase in use of psychotropic medications was noted over time, consistent with previous epidemiologic studies.^26^ The distribution of scanner field strengths differed among groups owing to a shift from 1.5- to 3-T clinical magnets in the late 2000s, a factor taken into account in the main analyses. Tagging patients’ last known addresses to block-level data obtained through the 2010 American Community Survey, we observed no differences among fortification groups in per capita use or spending on nonprescription vitamins, or in other measures that could affect folate intake (eg, median income, household educational level, or unemployment rate) (Table 1).

**Figure 1.**
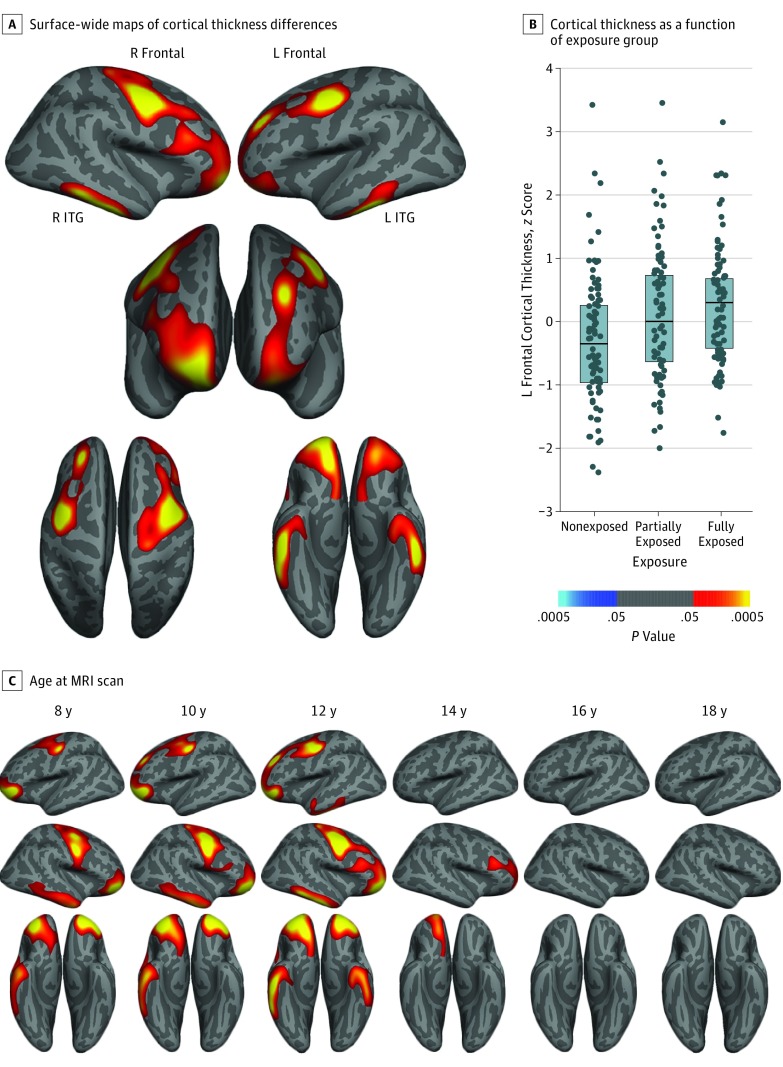
Fortification-Associated Cortical Thickness Changes in the Massachusetts General Hospital Cohort A, Surface-wide maps of cortical thickness in fully exposed (n = 99) minus nonexposed (n = 97) individuals reveal higher thickness among youths who were exposed to folic acid fortification during gestation. Images are masked to show only clusters that survive correction for multiple comparisons. B, Dot plots showing cortical thickness in the left frontal cluster as a function of exposure group, suggesting intermediate effects in the partially exposed group. Horizontal lines in the boxes indicate median values, and shaded boxes indicate interquartile ranges. Cortical thickness values are *z*-transformed residuals after controlling for nuisance covariates. Cool colors (shades of blue) show regions for which cortical thickness is greater in the group that was not exposed to fortification, whereas hot colors (red, orange, and yellow) show regions where cortical thickness is greater in the fully exposed group. C, Age-centered regression analyses indicate clusters with significant between-group differences in thickness as a function of age at magnetic resonance imaging (MRI) scan. This analysis indicates that overall group differences largely reflect exposure-related associations within younger individuals. ITG indicates inferior temporal gyrus; L, left, and R, right.

Group differences in cortical thickness were observed in bilateral frontal and inferior temporal regions. In each significant cluster, cortical thickness was higher in the fully exposed group compared with the nonexposed group, with intermediate effects observed in the partially exposed group (Figure 1A and B; eFigure 4 and eTable 3 in the Supplement). Sensitivity analyses revealed significant associations of scanner field strength and manufacturer with cortical thickness, but the direction of these associations varied by region, consistent with prior studies.^27,28^ Between-group differences remained significant after adjustment for these variables, and no significant group × field strength or group × manufacturer interactions were observed (eTable 4 in the Supplement).

**Figure 2.**
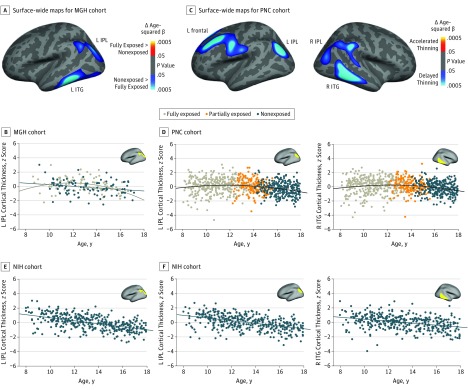
Fortification-Associated Emergence of Nonlinear (Delayed) Cortical Thinning A, Surface-wide maps of age-squared associations with cortical thickness in fully exposed (n = 99) minus nonexposed (n = 97) Massachusetts General Hospital (MGH) cohort scans reveal increased age-related quadratic thinning among individuals exposed to folic acid fortification during gestation. Nonexposed greater than fully exposed indicates that cool colors show regions for which β values are greater in the group that was not exposed to fortification. More negative β values reflect stronger quadratic thinning. The cool color (ie, shades of blue) reflects the negative age-squared term in the fully exposed group. B, Age-thickness scatterplot of MGH cohort, indicating emergence of quadratic (delayed) left inferior parietal lobule thinning in participants born after fortification was implemented. C, Surface-wide maps of age-squared associations with cortical thickness in the Philadelphia Neurodevelopmental Cohort (PNC) (n = 861) indicate age-related quadratic thinning in frontal, inferior parietal lobule (IPL), and inferior temporal gyrus (ITG) regions, again driven by delayed thinning in fully exposed individuals. D, Age-thickness scatterplot of the PNC cohort demonstrating delayed thinning in left (L) IPL and right (R) ITG. E, Within the L IPL cluster that demonstrated exposure-associated differences in quadratic thinning in the MGH cohort (A and B), analysis of the nonexposed National Institutes of Health Magnetic Resonance Imaging Study of Normal Brain Development (NIH) cohort indicates linear thinning (evident at the earliest time point). F, Similarly, within the L IPL and R ITG clusters that demonstrated quadratic thinning in the PNC cohort (C and D), only linear thinning was seen in the NIH cohort (383 scans). A and B, Images are masked to show only clusters that survive correction for multiple comparisons (*P* < .05, clusterwise; for PNC cohort L IPL, the displayed cluster was too small to survive correction at *P* < .05 but is significant at *P* < .01). Cortical thickness values in scatterplots represent *z*-transformed residuals after controlling for nuisance covariates.

To understand this pattern in the context of age-associated change in cortical thickness, we next assessed for group differences in intercept (ie, thickness means centered at 8 years of age) and slope (linear age effects), as well as for any differences in nonlinear (quadratic) age-associated change. Intercept in the bilateral frontal cortex (pars orbitalis and precentral) and the right inferior temporal gyrus was higher in the fully exposed group than in the nonexposed group; these differences diminished with age (Figure 1C; eFigure 5 and eTable 3 in the Supplement; and Video). There were no significant differences in the cortical thickness–age slope that contributed to the increased cortical thickness in the exposed groups. However, in the left inferior temporal cortex, as well as in the left inferior parietal cortex, we observed significant differences in age-squared effects, where cortical thinning in fully exposed participants was delayed compared with cortical thinning in nonexposed participants (Figure 2A and B; and eTable 5 in the Supplement). Additional modeling using least squares regression localized the onset of cortical thinning in fully exposed participants, defined by the optimal break point between flat and sloped lines, to 13.0 years of age (left inferior temporal gyrus) and 13.8 years of age (left inferior parietal lobule; eFigure 6A in the Supplement).

**Video.** Age-Related Group Differences in Cortical Thickness Lateral views of left and right cortex demonstrate dynamic effects of prenatal fortification exposure (fully exposed minus nonexposed) on cortical thickness between 8 and 18 years of age in the Massachusetts General Hospital cohort. In general, the most pronounced differences occurred at earlier ages. Color bar indicates effect size (Cohen *d*) and direction of effect.

### PNC and NIH Cohorts

The MGH data set relied on clinical MRI scans that were acquired through nonuniform clinical protocols, using different magnets, and within a single US city. To verify and generalize the findings from the MGH cohort, we next turned to 2 additional large US cohorts that were studied prospectively in standardized research settings (Table 2): 1 cohort with birthdates centered around the folic acid fortification rollout and MRI scans performed with a single 3-T magnet (PNC; eFigure 3 in the Supplement), and 1 cohort that included only youths who were born prior to folic acid fortification (NIH).

**Table 2.** Characteristics of PNC and NIH Cohort Participants

Characteristic	PNC Cohort (N = 861)					NIH Cohort (N = 217)
	Nonexposed (n = 322)	Partially Exposed (n = 189)	Fully Exposed (n = 350)	Statistics	*P* Value	
Age, mean (SD), y	16.3 (1.0)	13.9 (0.8)	10.7 (1.4)	*F* = 2032	<.001	13.3 (2.6)^a^
Female sex, No. (%)	183 (56.8)	95 (50.3)	166 (47.4)	χ^2^ = 6.10	.05	118 (54.4)
Race, No. (%)						
African American	132 (41.0)	89 (47.1)	151 (43.1)	χ^2^ = 5.35	.72	18 (8.3)
American Indian or Alaskan	0	1 (0.5)	1 (0.3)			0
Asian	4 (1.2)	1 (0.5)	6 (1.7)			1 (0.5)
White	152 (47.2)	77 (40.7)	151 (43.1)			171 (78.8)
Hawaiian or Pacific Islander	0	0	0			0
>1 Race	34 (10.6)	21 (11.1)	41 (11.7)			27 (12.4)
Ethnicity, No. (%) Hispanic	15 (4.7)	10 (5.3)	36 (10.3)	χ^2^ = 9.25	.01	29 (13.4)
Maternal educational level, No. (%)						
<High school graduate	13 (4.0)	8 (4.2)	18 (5.1)	χ^2^ = 15.1	.23	1 (0.5)
High school graduate	109 (33.9)	63 (33.3)	103 (29.4)			33 (15.2)
Some college or Associates degree	60 (18.6)	56 (29.6)	87 (24.9)			61 (28.1)
Bachelor’s degree	80 (24.8)	37 (19.6)	91 (26.0)			73 (33.6)
Some graduate education or Master’s degree	35 (10.9)	11 (5.8)	30 (8.6)			6 (2.8)
Graduate degree	13 (4.0)	8 (4.2)	13 (3.7)			41 (18.9)
Data not available	12 (3.7)	6 (3.2)	8 (2.3)			2 (0.9)
Paternal educational level, No. (%)						
<High school graduate	14 (4.3)	11 (5.8)	23 (6.6)	χ^2^ = 17.5	.13	5 (2.3)
High school graduate	117 (36.3)	74 (39.2)	136 (38.9)			42 (19.4)
Some college or Associates degree	45 (14.0)	36 (19.0)	58 (16.6)			52 (24.0)
Bachelor’s degree	67 (20.8)	23 (12.2)	59 (16.9)			62 (28.6)
Some graduate education or Master’s degree	25 (7.8)	15 (7.9)	34 (9.7)			7 (3.2)
Graduate degree	19 (5.9)	5 (2.6)	14 (4.0)			47 (21.7)
Data not available	35 (10.9)	25 (13.2)	26 (7.4)			2 (0.9)

Abbreviations: NIH, National Institutes of Health Magnetic Resonance Imaging Study of Normal Brain Development; PNC, Philadelphia Neurodevelopmental Cohort.

^a^ Mean age of participant across all scans, including up to 3 scans per participant.

Because MRI scans in the PNC data set were collected during a relatively brief time, nonexposed participants were significantly older than partially exposed or fully exposed participants. Whereas between-group cortical thickness differences would be strongly confounded by age differences, examination of age-associated thinning contours provided an opportunity to replicate findings from the MGH cohort. Specifically, younger PNC participants (who were exposed to folic acid fortification) should demonstrate delayed cortical thinning, whereas younger NIH participants (who were not exposed to folic acid fortification) should not demonstrate delayed cortical thinning.

Within the PNC cohort, quadratic (delayed) age-related thinning was observed in 4 clusters that overlapped with those identified in the MGH analysis: left frontal, right inferior temporal, left inferior parietal, and right inferior parietal (Figure 2C and D; and eFigure 7A and B and eTable 6 in the Supplement). Least squares regression localized the onsets of cortical thinning to between 13.0 and 14.3 years of age (eFigure 6B in the Supplement). Conversely, bilateral lingual gyrus (which was not implicated in the MGH cohort) exhibited significantly accelerated cortical thinning.

To confirm that quadratic thinning effects emerged largely after folic acid fortification, we turned to the NIH cohort, in which all included participants were born prior to the rollout. Age-related associations with cortical thickness were assessed within the same 6 clusters that showed delayed thinning in the MGH (left inferior temporal gyrus and left inferior parietal lobule) or PNC (right inferior temporal gyrus, left frontal cortex, left inferior parietal lobule, and right inferior parietal lobule) cohorts. Consistent with a previous analysis^11^ that included a more extended age range and that was not limited to nonexposed participants, nonlinear cortical thinning in the NIH cohort was sparse, with only 1 cluster demonstrating significant quadratic thinning (left frontal cortex; Figure 2E and F; and eFigure 7C and eTable 7 in the Supplement). Even so, within this cluster, the break point for thinning occurred at a significantly younger age when compared with the PNC break point (χ^2^ = 11.87; *P* < .001; eFigure 6C in the Supplement).

### Risk of Psychosis

**Figure 3.**
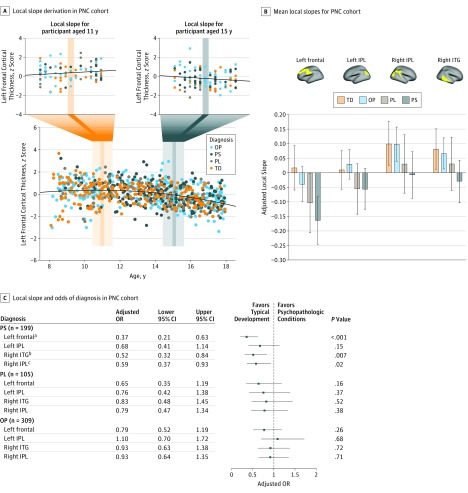
Local Slope Derivation and Association of Delayed Cortical Thinning With Individual Risk for Psychopathologic Conditions in Participants in the Philadelphia Neurodevelopmental Cohort (PNC) A, In each of the 4 clusters exhibiting delayed cortical thinning in the PNC cohort, local cortical thinning slope was calculated for each individual based on the best-fit line of thickness vs age among all nearby individuals (±6 months). Insets demonstrate local slopes for an 11.0-year-old (left) and a 15.0-year-old (right) participant. B, Mean local slopes for participants in each diagnostic group, in each of the 4 clusters. For example, individuals with psychosis spectrum (PS) symptoms tended to have more negative slopes than those with other diagnoses. Local slopes reflect change in z-transformed cortical thickness scores (adjusted for nuisance covariates) during 1 year. C, Multinomial logistic regression models associated with diagnosis (PS, psychosis low [PL], or other psychopathologic condition [OP] relative to typically developing [TD]; n = 248) of each participant based on local slope, covarying for age, sex, total brain volume, and method of ascertaining diagnosis. Lower adjusted odds ratios indicate reduced odds of psychopathologic condition in the presence of flatter (less negative) local thinning slope, a pattern that was significant for PS in 3 of 4 regions tested. All *P* values are false discovery rate corrected. All error bars indicate 95% CIs. IPL indicates inferior parietal lobule; ITG, inferior temporal gyrus. ^a^*P* < .001. ^b^*P* < .01. ^c^*P* < .05.

To gauge the association of altered cortical thinning with clinically relevant phenotypes, we again turned to the PNC cohort, which included a detailed, standardized clinical characterization of all participants. Of the 861 youths included in the MRI analysis, clinical evaluations determined that 248 were typically developing, 199 had a diagnosis of psychosis spectrum, 105 had attenuated psychotic symptoms, and the remaining 309 had various other psychopathologic conditions, as previously described (eAppendix in the Supplement).^23,24,29^ For each of the 4 PNC regions that demonstrated postfortification quadratic thinning, best-fit local thinning slopes were calculated for each participant, based on linear change in cortical thickness across an optimized age range (1 year) centered around that participant (Figure 3A). These local slopes were then evaluated as factors associated with participant-level diagnosis of psychosis spectrum, attenuated psychosis, or other types of psychopathologic conditions vs typical development using multinomial logistic regression, controlling for nuisance covariates. Across 3 of 4 regions, flatter (ie, less negative) local slopes were associated with significantly reduced adjusted odds of psychosis spectrum diagnosis (odds ratio, 0.37-0.59; *P* < .001 to *P* = .02, Figure 3B and C). Local slopes were not associated with risk of other types of psychopathologic conditions in any region. For participants with attenuated psychosis, local slope associations were stronger than for other types of psychopathologic conditions, but nonsignificant.

Finally, to confirm that the associations of fortification exposure with cortical thinning were not themselves confounded by the inclusion of individuals with psychosis spectrum symptoms in the PNC cohort, we repeated the original analysis of quadratic thinning using only typically developing individuals or participants with other types of psychopathologic conditions (n = 541). Significant quadratic age-associated thinning persisted in all regions after exclusion of individuals with psychosis spectrum symptoms and youths with attenuated psychosis (eTable 8 in the Supplement).

## Discussion

Evaluating 3 independent MRI cohorts of 8- to 18-year-old youths, we investigated the association of prenatal exposure to folic acid fortification with subsequent cortical development through adolescence. Within a large clinical cohort (MGH), we observed widespread increases in frontal and temporal cortical thickness between comparable groups of youths who gestated just after, compared with just before, the rollout of folic acid fortification. Youths who gestated during the rollout, and who therefore had partial exposure, demonstrated intermediate increases, consistent with a dose association. Exposure-associated differences were most pronounced in younger individuals, and in 2 regions (left inferior temporal and inferior parietal), we observed a delay in the onset of age-associated cortical thinning. An analogous pattern was evident in the contemporaneous PNC cohort: individuals who were exposed to folic acid fortification exhibited delays of cortical thinning of similar duration, which occurred in similar frontal, temporal, and parietal regions as those identified in the MGH cohort. Flatter age-related thinning profiles were associated with reduced risk of psychosis spectrum symptoms in this cohort. In contrast, the NIH cohort, comprising only individuals who were not exposed to fortification, exhibited earlier cortical thinning in the same regions. Collectively, these data suggest an association of prenatal exposure to folic acid fortification with increased cortical thickness through early adolescence, accompanied by delayed onset of cortical thinning and reduced risk of psychosis.

Adolescence directly precedes the period of greatest risk for psychiatric disorders, some of which are characterized by reductions in cortical thickness present at the onset of illness.^30^ Furthermore, some of the most severe child-onset psychiatric disorders, including autism and early-onset schizophrenia, are associated with marked accelerations in loss of gray matter during the age range that we studied.^16,18^ Within the PNC cohort, reductions in gray matter in multiple brain regions were associated with psychosis spectrum status in a previous analysis conducted without regard to exposure to folic acid fortification^24^; however, these regions differed from those demonstrating fortification-associated thinning delays herein. Rather, within these regions, shallower thinning slopes were associated with reduced risk for psychosis spectrum symptoms, suggesting a possible protective effect of fortification-associated delays in cortical thinning. This association was relatively specific for psychosis because local slopes were not associated with other psychopathologic conditions, and attenuated associations were seen in individuals with milder psychotic spectrum symptoms. The present findings are consistent with recent reports of salutary behavioral outcomes after periconceptional intake of folic acid^6,7,8,9,31^ and with a recent study linking maternal folate deficiency with reduced brain volume in a cohort of young European children.^32^

### Limitations

Although relatively large imaging cohorts, replication and temporal specificity analyses using independent samples, and linkage of imaging and clinical findings represent the strengths of our study, a number of potential limitations warrant consideration. Unrecognized temporal confounders are of particular concern in natural experimental designs. Critically, the rapid deployment of folic acid fortification in the United States allowed us to study a relatively narrow and continuous range of birthdates, which diminished the risk of potential temporal confounders, including group differences in postnatal exposure to folic acid fortification. We also sought to address a number of other potential clinical, demographic, and socioeconomic confounders through extended use of electronic medical records and block-level American Community Survey data, but we saw no substantial differences among exposure groups. Although, on the population level, the rollout of folic acid fortification rapidly doubled blood folate levels without changing folic acid supplement (vitamin) use,^2,33^ the lack of individual-level data on maternal folate intake represents an inevitable limitation of this experiment’s design. However, exposure groups were comparable on block-level vitamin spending and consumption.

Although exposure groups in the MGH cohort were generally well matched, we cannot rule out the potential role of magnetic field strength differences within that cohort. Field strength effects would not be expected to bias results consistently because previous work indicates that the associations of field strength with cortical thickness measurements vary in direction and magnitude across the cortex^27^; recognizing this heterogeneity, field strength was entered as a covariate in the surface-wide analysis. Furthermore, within regions demonstrating between-group differences, these differences remained significant in sensitivity analyses that controlled for scanner field strength and manufacturer. Perhaps more important, that similar exposure-related associations with cortical thinning were observed in the PNC cohort, which used a single 3-T magnet, suggests that scanner differences did not substantially influence the MGH cohort results. More broadly, similar findings across 2 cohorts that differed substantially in terms of population sampling strategy (retrospective and clinical vs prospective and community-based), imaging and clinical assessment (medical record–based vs standardized), and geographic location—but that were comparable in terms of exposure, sex, age, race/ethnicity, and urbanicity—suggest reliability of the findings.

The present findings also raise questions about the long-lasting effects of variation in the fetal methylome because folate supplies 1-carbon moieties that regulate gene expression. One possibility suggested by the present results is that programming of cortical maturation in youths is sensitive to fetal folate levels, potentially via epigenetic modification of genes that regulate cortical development,^34^ repair of de novo mutations,^35^ or mitigation of toxic exposures.^36,37,38,39^ Although, to our knowledge, these results are the first to link prenatal exposure to folic acid fortification to changes in subsequent cortical development, the specific mechanisms underlying these effects have yet to be elucidated.

## Conclusions

In replicated cohorts, fetal exposure to population-wide folic acid fortification was associated with subsequent alterations in cortical development among school-aged youths. In turn, these cortical changes were associated with reduced risk of psychosis. Our findings suggest that protective effects of prenatal folic acid exposure may extend beyond prevention of neural tube defects and span neurodevelopment during childhood and adolescence.

## Notes

Supplement. **eAppendix.** Methods **eFigure 1.** Countries and Territories With Mandatory Folic Acid Fortification of Grain Products, as of November 2017 **eFigure 2.** Medical Record Search Algorithm and Image Processing Pipeline for the MGH Cohort **eFigure 3.** Distribution of Birth Year Among MGH and PNC Cohort Individuals **eFigure 4.** Additional Clusters Demonstrating Exposure-Related Effects on Cortical Thickness in the MGH Cohort **eFigure 5.** Comparison of Significant Mean Age-Centered and Intercept (Age 8)–Centered Clusters Showing Exposure-Related Effects in the MGH Cohort **eFigure 6.** Estimate of Thinning Delay in MGH and PNC Clusters Showing Quadratic Age-Related Thinning **eFigure 7.** Additional Clusters Demonstrating Quadratic Age-Related Thinning in the PNC Cohort **eTable 1**. Radiologic Exclusion Criteria for MGH Cohort **eTable 2**. Comparison of Included And Excluded Individuals in MGH Cohort **eTable 3**. Clusters Demonstrating Significantly Increased Cortical Thickness in Fully Exposed Versus Non-Exposed Youths in MGH Cohort **eTable 4.** Effects of Scanner Strength and Manufacturer Differences on Group Cortical Thickness Differences in MGH Cohort **eTable 5.** Clusters Demonstrating Significantly Different Quadratic Age-Related Thinning in Fully Exposed Versus Non-Exposed Youths in MGH Cohort **eTable 6.** Clusters Demonstrating Quadratic Age-Related Thinning in PNC Cohort **eTable 7.** Tests for Quadratic Age-Related Thinning in NIH Cohort, Within Clusters Demonstrating Significant Quadratic Thinning in MGH or PNC Cohorts **eTable 8.** Quadratic Age-Related Thinning in PNC Cohort Before and After Exclusion of Youths With Psychotic Symptoms **eReferences**
