# Supplementary material for: Association of Prenatal Exposure to Population-Wide Folic Acid Fortification With Altered Cerebral Cortex Maturation in Youths
Source: JAMA Psychiatry. 2018 Jul 3;75(9):918–28. doi: 10.1001/jamapsychiatry.2018.1381 (PMC6142921; doi:10.1001/jamapsychiatry.2018.1381)
Supplement: Supplement. — eAppendix. Methods eFigure 1. Countries and Territories With Mandatory Folic Acid Fortification of Grain Products, as of November 2017 eFigure 2. Medical Record Search Algorithm and Image Processing Pipeline for the MGH Cohort eFigure 3. Distribution of Birth Year Among MGH and PNC Cohort Individuals eFigure 4. Additional Clusters Demonstrating Exposure-Related Effects on Cortical Thickness in the MGH Cohort eFigure 5. Comparison of Significant Mean Age-Centered and Intercept (Age 8)–Centered Clusters Showing Exposure-Related Effects in the MGH Cohort eFigure 6. Estimate of Thinning Delay in MGH and PNC Clusters Showing Quadratic Age-Related Thinning eFigure 7. Additional Clusters Demonstrating Quadratic Age-Related Thinning in the PNC Cohort eTable 1. Radiologic Exclusion Criteria for MGH Cohort eTable 2. Comparison of Included And Excluded Individuals in MGH Cohort eTable 3. Clusters Demonstrating Significantly Increased Cortical Thickness in Fully Exposed Versus Non-Exposed Youths in MGH Cohort eTable 4. Effects of Scanner Strength and Manufacturer Differences on Group Cortical Thickness Differences in MGH Cohort eTable 5. Clusters Demonstrating Significantly Different Quadratic Age-Related Thinning in Fully Exposed Versus Non-Exposed Youths in MGH Cohort eTable 6. Clusters Demonstrating Quadratic Age-Related Thinning in PNC Cohort eTable 7. Tests for Quadratic Age-Related Thinning in NIH Cohort, Within Clusters Demonstrating Significant Quadratic Thinning in MGH or PNC Cohorts eTable 8. Quadratic Age-Related Thinning in PNC Cohort Before and After Exclusion of Youths With Psychotic Symptoms eReferences [file jamapsychiatry-75-918-s001.pdf]

Supplementary Online Content

Eryilmaz H, Dowling KF, Huntington FC, et al. Association of prenatal exposure to population-wide folic acid fortification with altered cerebral cortex maturation in youths. *JAMA Psychiatry.* Published online July 3, 2018. doi:10.1001/jamapsychiatry.2018.1381

eAppendix. Methods
eFigure 1. Countries and Territories With Mandatory Folic Acid Fortification of Grain Products, as of November 2017
eFigure 2. Medical Record Search Algorithm and Image Processing Pipeline for the MGH Cohort
eFigure 3. Distribution of Birth Year Among MGH and PNC Cohort Individuals
eFigure 4. Additional Clusters Demonstrating Exposure-Related Effects on Cortical Thickness in the MGH Cohort
eFigure 5. Comparison of Significant Mean Age-Centered and Intercept (Age 8)–Centered Clusters Showing Exposure-Related Effects in the MGH Cohort
eFigure 6. Estimate of Thinning Delay in MGH and PNC Clusters Showing Quadratic Age-Related Thinning
eFigure 7. Additional Clusters Demonstrating Quadratic Age-Related Thinning in the PNC Cohort
eTable 1. Radiologic Exclusion Criteria for MGH Cohort
eTable 2. Comparison of Included and Excluded Individuals in MGH Cohort
eTable 3. Clusters Demonstrating Significantly Increased Cortical Thickness in Fully Exposed Versus Nonexposed Youths in MGH Cohort
eTable 4. Effects of Scanner Strength and Manufacturer Differences on Group Cortical Thickness Differences in MGH Cohort
eTable 5. Clusters Demonstrating Significantly Different Quadratic Age-Related Thinning in Fully Exposed Versus Nonexposed Youths in MGH Cohort
eTable 6. Clusters Demonstrating Quadratic Age-Related Thinning in PNC Cohort
eTable 7. Tests for Quadratic Age-Related Thinning in NIH Cohort, Within Clusters Demonstrating Significant Quadratic Thinning in MGH or PNC Cohorts
eTable 8. Quadratic Age-Related Thinning in PNC Cohort Before and After Exclusion of Youths With Psychotic Symptoms
eReferences

This supplementary material has been provided by the authors to give readers additional information about their work.

© 2018 Eryilmaz H et al. *JAMA Psychiatry.*

eAppendix. Methods

Sources of data. We studied 3 independent cohorts in the current investigation. The first dataset (MGH) was acquired from the Partners Research Patient Data Registry (RPDR), a central clinical data warehouse containing the electronic health record data for more than 6.5 million patients in the Partners HealthCare Network. Study procedures were approved by the Partners HealthCare Human Research Committee (IRB), which granted a waiver of informed consent for this retrospective study of the medical record. Using the RPDR Query Tool, radiology reports were obtained for patients meeting general inclusion criteria (age 8.0 to 18.0, receiving non-contrast brain MRI scans at MGH between January 2005 and March 2015). A pre-determined algorithm sampled this cohort to generate age-matched groups of patients born before (1/1/93-6/30/96), during (7/1/96-6/30/98), or after (7/1/98-12/31/01) the fortification rollout as described in greater detail below and in **eFigure 2a**. Power analysis of pilot data from pre- and post-fortification scans (N=45) indicated a minimum detectible difference of 0.10 to 0.13 mm (depending on region) with 80% power at alpha=.05, based on 50 subjects per group.

The second dataset (PNC) involved 8- to 21-year-old youths who were randomly selected from primary care clinics in the Philadelphia area and who received prospective, standardized MRI scans[1]. IRB approval and informed consent have been described elsewhere[1]. As part of this initiative, 1,445 participants underwent standardized MRI scans. All participants were scanned using a single 3T Siemens Trio magnet located in the Hospital of the University of Pennsylvania. Image quality was assessed by rigorous visual inspection, which primarily eliminated scans with artifacts due to head motion[2]. 1,332 subjects passed the visual quality control. We selected a subsample from this group that was constrained to our age group of interest (8.0-18.0, based on age at scan in months), and eliminated scans with major medical problems that could impact brain function[3]. Scans missing total brain volumes were also excluded. The final PNC sample consisted of 861 individuals, scanned from March, 2010 to July, 2012. Birth dates for the PNC group were centered around the rollout of folic acid fortification, which facilitated the replication of thinning profiles from the MGH dataset (see **eFigure 3**)[1].

MRI scans from a third cohort (NIH), obtained from the NIH MRI Study of Normal Brain Development[4], were used to study thinning trajectories in a sample of deidentified youth who gestated prior to the folate rollout. IRB approval and informed consent have been described elsewhere[5]. This project recruited a total of 431 healthy subjects aging between 4.6 and 18 years (at the time of first enrollment) in 6 pediatric study centers in the US between November 2001 and September 2003. Participants underwent MRI scans in 1.5T GE and Siemens scanners. Data collection for follow up scans continued until November 2007. For our study, we selected a subsample from this cohort that was previously identified as passing stringent quality control measures[6], and further narrowed it to our age interval of interest (8.0 to 18.0). In order to ensure that none of the subjects in our subsample was exposed to folic acid fortification we only included subjects that were 87 months and older at the time of first enrollment. The final sample included 217 individual subjects and 383 scans.

Medical record sampling in the MGH cohort. Using RPDR the MGH electronic medical record was searched using a pre-determined rubric to obtain age-matched groups of youth, born before (1/1/93-6/30/96), during (7/1/96-6/30/98), or after (7/1/98-12/31/01) the fortification rollout, and who as 8.0- to 18.0-year-olds had undergone clinical brain MRI scans at MGH 2005 to 2015 (**eFigure 2a,b**). Radiology reports (n=3,311) were returned by RPDR and manually examined for exclusion criteria (patient deceased since scan (n=51); patient born outside US or no address given (n=152); patient diagnosed with prohibitive comorbidity or image quality issues (n=1,997); see **eTable 2**). Accordingly, 2,200 reports screened out.

MRI quality control and final scan selection in the MGH cohort. MRI data for the remaining reports (n=1,111) were then visually inspected for image quality by a trained investigator using ChRIS software[7]. Image inspection was conducted blind to group assignment. 796 scans were excluded due to the failure to meet stringent quality control (presence of any visible motion, susceptibility, or other artifact; poor contrast; ghosting; ringing; aliasing; any blurring of gray-white matter demarcation), by the absence of high-resolution T1-weighted structural scans conducted at MGH, or by the presence of more than one scan per patient (in which case only the first scan was used).

© 2018 Eryilmaz H et al. *JAMA Psychiatry.*

The remaining 315 scans were pre-processed using FreeSurfer v5.0, which provides an automated segmentation of cortical gray matter. Each of the pre-processed scans were then subjected to manual editing of the pial and gray-white boundaries[8,9] by a single technician who was blind to all subject-level information (including exposure group). An additional 23 scans were excluded during editing due to skull-wrap deformities or other non-editable abnormalities. The remaining 292 scans were included in the analysis. These scans were obtained at various imaging sites at MGH using one of the five scanner types: 1.5T GE, 1.5T Siemens Avanto, 1.5T Siemens Aera, 3T Siemens Trio, and 3T Siemens Skyra. Subjects were sorted into pre-rollout (nonexposed, n=97), rollout (partially exposed, n=96) and post-rollout (fully exposed, n=99) groups based on their date of birth (**eFigure 2b**). This sample size exceeded that which was pre-determined to be sufficiently powered for group cortical thickness analyses, as above.

Geospatial analysis of socioeconomic variables and vitamin intake. Addresses from all 292 MGH patients were geocoded with the Esri Business Analyst 2015 address locator. All addresses were located to the address level of accuracy. The address locations were then overlaid with the Business Analyst 2010 block group dataset, and corresponding economic, education, and 6-month consumer vitamin use and spending variables were extracted from the block group each address fell into. Block groups are subdivisions of census tracks and provide neighborhood-level analysis of approximately 700 residents. Five addresses could not be located within block groups; for these subjects, zip code-level data were used. Vitamin use data is reported as an index value, which may be compared to nationwide average value of 100.

Image processing. All scans in this study were analyzed using FreeSurfer v5.0 (http://surfer.nmr.mgh.harvard.edu/). The automated image processing pipeline of FreeSurfer performs full characterization of the anatomy including the cortex, white matter/gray matter boundaries, folding patterns, cortical and subcortical regions of interest. Specifically, the typical processing of a structural MR image includes removal of non-brain tissue, automated Talairach transformation, segmentation of the subcortical white matter and deep gray matter structures, intensity normalization, tessellation of the gray/white matter boundary, automated topology correction, and surface deformation following intensity gradients to optimally position the gray/white and gray/cerebrospinal fluid (CSF) borders[8,10]. Once the cortical model is complete, the cerebral cortex is parcellated into units based on the gyral and sulcal structure[11]. This method uses both intensity and continuity information from the volumetric image to produce representations of cortical thickness, calculated as the closest distance from the gray/white boundary to the gray/CSF boundary at each vertex on the tessellated surface[10]. Methods for cortical thickness measurements have been validated against histological analyses[12] and manual measurements[13]. Importantly, FreeSurfer structural image processing has been demonstrated to show high test-retest reliability across scanner manufacturers and across field strengths[14,15]. In all imaging analyses, individual thickness maps were smoothed with a 22-mm full width half maximum (FWHM) kernel in order to remove noise-related variations in the images.

Cortical thickness analyses in MGH cohort. To contrast thickness in nonexposed and fully exposed groups, cortical surface clusters were identified based on significant group differences, defined by a cluster extent threshold $p<.05$ and cluster-wise correction for multiple comparisons across the entire cortical surface using 10,000 Monte Carlo simulations (cluster-wise $p<.05$). Z-transformed age, age^2, sex, total brain volume (TBV), and scanner field strength, which have been shown to influence cortical thickness[6,16] were entered as nuisance covariates. Of note, both scanner field strength and manufacturer have been associated with effects on cortical thickness measurements (albeit in different directions depending on cortical location)[14,16] . Within the MGH sample, all GE scans were conducted at 1.5T, while Siemens scans were conducted at 1.5 and 3T. Given that scanner field strength and manufacturer were highly collinear, and that field strength has been associated with stronger effects on cortical thickness than manufacturer [14], we chose (a priori) field strength rather than manufacturer as a covariate. While each of the five scanner platforms used slightly different T1 acquisition sequences, given the uneven distribution of scanners across the sample (**Table 1**), it would have been impractical to include specific scanner platform as a covariate, over and above scanner field strength.

After significant clusters were defined in the group analysis, individual-level thickness values (indicating mean thickness across all vertices within the group-average cluster) were extracted for those clusters in each subject in the nonexposed, partially exposed, and fully exposed groups. These values were used to generate subject-level plots (e.g., **Figure 1b, 2b**) and to further quantify group differences in terms of percent difference (**eTable 3**). Additional sensitivity analyses (MANCOVA) examined effects of both scanner field strength and scanner manufacturer on cortical thickness in regions demonstrating significant between-group differences.

© 2018 Eryilmaz H et al. *JAMA Psychiatry.*

To understand exposure-related effects within the context of brain development, additional surface-wide analyses probed group differences in age-related cortical thinning contours (i.e., intercept, slope, and non-linear thinning). First, difference maps for nonexposed versus fully exposed groups were re-centered at all ages from 8.0 to 18.0 by shifting subject ages in 0.1 y increments (raw values, not z-transformed) accordingly and introducing an age x group covariate into the general linear model. At each age (e.g., 8.0), Monte Carlo analysis was repeated to identify significant between-group differences in cortical thickness at that age point (e.g., **Figure 1c, Video**). Second, to determine whether and where differences in age-thickness slope contributed to overall group thickness differences, the age x group interaction term was selected as the covariate of interest (i.e., with z-transformed age, group, sex, scanner strength, and TBV as nuisance covariates). Finally, to determine if groups differed in non-linear thinning contours, age^2 x group was entered as the covariate of interest, with nuisance covariates as above.

Cortical thickness analyses in PNC cohort. Surface-wide analysis of non-linear thinning was assessed across the entire PNC sample. The general linear model included age^2 as the covariate of interest, and z-transformed age, sex, and TBV as nuisance covariates. Scanner strength was not entered into the model as all participants were scanned on the same 3T magnet. As above, clusters were defined as significant based on a cluster extent threshold $p<.05$ and surface-wide correction for multiple comparisons using 10,000 Monte Carlo simulations (cluster-wise $p<.05$). Subject-level data on cortical thickness within significant clusters were extracted as above.

Cortical thickness analyses in the NIH cohort. To determine whether non-linear thinning occurred prior to the fortification rollout, clusters identified as having quadratic age-thinning contours in the MGH or PNC cohorts were assessed for non-linear thinning in the NIH cohort. As the NIH sample comprised subjects with between one and three scans, linear mixed models were employed (SPSS v25), using a diagonal covariance structure. Individual-level thickness values were extracted from each cluster (as above) and were treated as the dependent variable. Subject ID and visit number were entered as repeated measures; sex and scanner site were entered as categorical covariates; and z-transformed TBV, age, and age^2 were entered as continuous covariates. Clusters where age^2 predicted cortical thickness at $p<.05$ (uncorrected) were considered to show significant quadratic thinning.

Break point analyses for regions demonstrating quadratic thinning. For regions demonstrating quadratic (delayed) age-related thinning in the MGH, PNC, or NIH cohort, we estimated the break point (age in years) where the age-thickness relationship changed from flat to sloped using least squares analysis (e.g., for **eFigure 6**). Subject-level cortical thickness values were extracted from the cluster (as above); sex, TBV, and other cohort-specific nuisance covariates (scanner strength for MGH, site for NIH) were regressed out, and z-transformed residual cortical thickness values were used for subsequent analysis (SPSS v25). Using Matlab R2015b (https://www.mathworks.com), break points were tested serially, starting at age 9.0 and then incrementally increasing by 0.1 year at a time up to 17.0. Break point range was determined as 9.0-17.0 to ensure sufficient data for the computation of goodness-of-fit at both ends. At a given break point, age-related thinning changes were modeled by a "flat-slope" function as follows: (1) the average of all thickness values from age 8.0 up to and including the break point became the y-value for the "flat" part of the thickness vs. age function, and (2) the best fit line of all remaining thickness values occurring after the break point, constrained by having left edge of the new sloped line contiguous with the flat line established in step 1, became the "slope" part of the thickness vs. age function. At each 0.1-year break point interval, we determined and squared the vertical distance between each cortical thickness data point and the "flat-slope" function derived for that interval; the average of these values became the mean of squares for that particular break point, reflecting the goodness-of-fit of that break point model with respect to the actual data from participants aged 8 to 18. We then plotted the average of squares as a function of break point age, generating an inverted-U shaped curve, where the minimum value of the curve identified the break point with optimal fit.

In the case of the NIH cohort cluster showing quadratic thinning (i.e., where the cluster was defined based on quadratic thinning in the MGH or PNC cohorts, and then found to also be quadratic in the NIH cohort), we determined whether the break point defined through the analysis of NIH data differed significantly from the break point defined through the referring data set (MGH or PNC) using chi-square. For example, if the break point for a given cluster occurred at age 10.0 in the PNC data and at 12.0 in the NIH data, chi-squared was conducted using the NIH data, and determined whether the proportion of subjects age 10 and under differed significantly from the proportion of subjects age 12 and under.

© 2018 Eryilmaz H et al. *JAMA Psychiatry.*

Clinical phenotyping of PNC participants. PNC participants received standardized clinical evaluations using a structured computerized screening instrument (GOASSESS) as previously described[17-19]. Psychiatric history was obtained from a caregiver (participants age 8 to 10) or caregiver and proband (participants age 11 to 18). Psychosis Spectrum youth (PS) were identified based on (1) an age-deviant score of ≥2 SD above age-matched peers on the Prevention through Risk Identification, Management, and Education (PRIME) Screen-Revised (PS-R)[20], or the presence of ≥1 PRIME item rated 6 or ≥3 items rated at least 5; (2) endorsed definite or possible hallucinations on the Kiddie Schedule for Affective Disorders and Schizophrenia (K-SADS)[21] psychosis screen; or (3) had an age-deviant total negative/disorganized Scale of Prodromal Symptoms (SOPS)[22] score ≥2 SD above age-matched peers. Psychosis Low youth (PL) included subjects who endorsed more subpsychotic (PS-R) or subthreshold negative or disorganized (SOPS) symptoms than their age-matched peers (≥1 SD) but who did not meet full criteria for PS. Typically Developing (TD) youth lacked any significant psychopathology, history of psychotropic medication use, or history of inpatient psychiatric hospitalization. Remaining youth were classified as Other Psychopathology (OP), indicating significant non-psychotic symptoms (mood, anxiety, attention-deficit, disruptive behavior, or eating disorders) and/or psychotropic medication use. Of the 881 included PNC participants, 209 were classified as TD, 216 as PS, 111 as PL, and the remaining 345 as OP. A total of 30 participants in the OP group, 43 in the PS group, and 19 in the PL group had a history of exposure to psychotropic medications. The prevalence of psychosis spectrum symptoms observed in the PNC sample[19] is consistent with previous studies investigating the similar age range. A meta-analysis of population-based studies in children and adolescents showed higher rates of psychotic-like experiences in youth than in adults (range 5-35%), with meta-analytically derived medians of 17% in children (9-12 years old), and 7.5% in adolescents (13-18 years old)[23].

Derivation of local cortical thinning slopes in PNC participants. To test the hypothesis that cortical thinning delays related to fortification exposure conferred protection against psychosis risk, we first needed to derive a subject-level index for whether individuals were situated in relatively flat or steep parts of the age-related thinning curve. As such, best-fit local thinning slopes for cortical thickness versus age were calculated for each subject. Subject-level cortical thickness values were extracted from the cluster as above; sex and TBV were regressed out, and z-transformed residual cortical thickness values were used for subsequent analysis (SPSS v25). Before testing the effects of local best-fit slopes on clinical phenotypes, the method for obtaining these slopes was optimized by varying the temporal window size, i.e., the number of months' worth of data on either side of a given subject to include for that subject's local slope measurement. Data on the extreme ends were excluded (e.g., for a temporal window of six months, only subjects between 8.5 and 17.5 were included). In selecting the optimal window size, normality (Kolmogorov-Smirnov test) of local slope distributions was the primary metric of interest, and slope and kurtosis were secondary metrics of interest. For regions identified as showing significant quadratic thinning in the PNC cohorts, empirical tests of these parameters for various window sizes are summarized below.

Region	± 3 month window			± 6 month window			± 12 month window			± 24 month window		
	Norm.	Skew	Kurt.	Norm.	Skew	Kurt.	Norm.	Skew	Kurt.	Norm.	Skew	Kurt.
L Frontal	0.092	1.140	3.500	0.099	0.371	1.750	0.131	0.838	0.398	0.200	0.286	-1.647
L IPL	0.085	0.190	-0.346	0.056	-0.217	-0.658	0.116	0.251	-1.048	0.233	0.557	-1.351
R IPL	0.073	0.776	3.851	0.067	0.083	0.276	0.097	0.594	-0.116	0.185	0.296	-1.575
R ITG	0.052	0.395	2.033	0.067	0.226	0.192	0.078	0.618	-0.150	0.212	0.540	-1.306
**Average**	**0.076**	**0.625**	**2.260**	**0.072**	**0.116**	**0.390**	**0.106**	**0.575**	**-0.229**	**0.208**	**0.420**	**-1.470**

L, left; R, right; IPL, inferior parietal lobule; ITG, inferior temporal gyrus

An inclusion window of six months on either side of the subject was found to be optimal, and therefore this window size was used in the computation of local slopes.

Relation of local cortical thinning slopes to clinical phenotypes in PNC participants. Multinomial logistic regression models (SPSS v25) were used to determine whether local thinning slopes in clusters with significant quadratic (delayed) thinning predicted diagnosis, with one model per region (4 total analyses). PS, PL, and OP were each compared to TD within the same multinomial model. A priori covariates included age and method of diagnosis ascertainment [binary, based on history from caregiver only (age 8-10) versus caregiver plus proband (age 11-18)], as well as sex and TBV, given established relationships between these factors and psychosis risk. Resulting adjusted odds ratios reflect the relationship between subjects' local thinning slopes and odds of diagnosis (PS, PL, or OP

© 2018 Eryilmaz H et al. *JAMA Psychiatry.*

versus TD). The false discovery rate (alpha=.05) was used to correct for analyses in 4 regions, as thickness values across regions were not independent of each other.

© 2018 Eryilmaz H et al. *JAMA Psychiatry.*

eFigure 1. Countries and Territories With Mandatory Folic Acid Fortification of Grain Products, as of November 2017

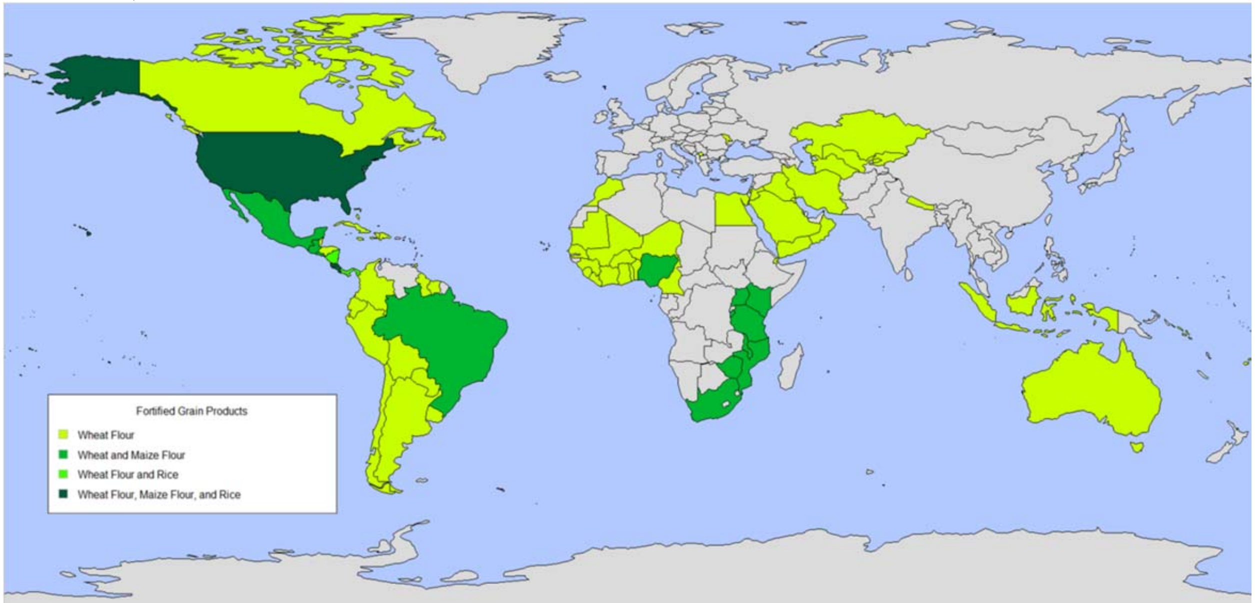

A total of 81 countries and territories, comprising 2.4 billion individuals, require some degree of food fortification with folic acid. Data were accessed from the Food Fortification Initiative (http://www.ffinetwork.org/country_profiles/index.php) on 21 January 2018. According to United Nations estimates (https://esa.un.org/unpd/wpp/), 55 million children are born annually in countries that require fortification. National fortification policies differ somewhat according to local dietary staples (see Legend). Map generated in R (www.R-project.org) using the rworldmap package.

© 2018 Eryilmaz H et al. *JAMA Psychiatry.*

eFigure 2. Medical Record Search Algorithm and Image Processing Pipeline for the MGH Cohort

A

Birth year	Year of scan: 2005	2006	2007	2008	2009	2010	2011	2012	2013	2014	2015
	Age at scan										
1993	12	13	14								
1994	11	12	13	14	15						
1995	10	11	12	13	14	15	16				
1996	9	10	11	12	13	14	15	16	17		
1997		9	10	11	12	13	14	15	16	17	
1998			9	10	11	12	13	14	15	16	17
1999					10	11	12	13	14	15	16
2000							11	12	13	14	15
2001									12	13	14

B

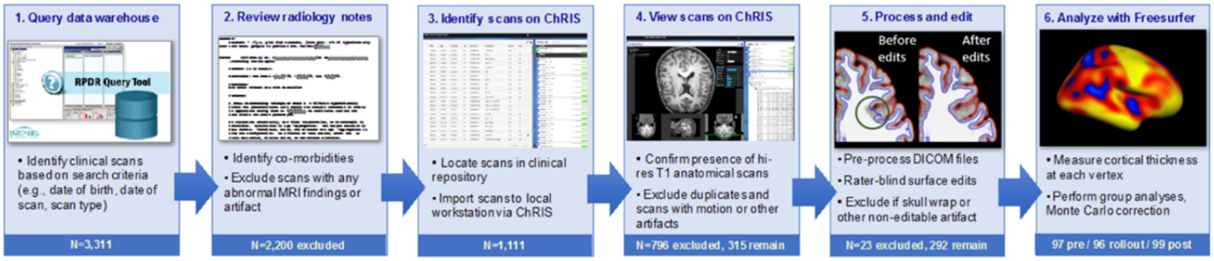

(A) Using the Partners HealthCare Research Patient Data Registry, we purposely sampled the medical record to identify age-matched groups of youth, age 8.0 to 18.0, who gestated before (blue), during (pink), or after (green) the folic acid fortification rollout and who underwent a clinical MRI scan at MGH. For example, to populate the upper left-most cell, we searched for youth who were born in 1993 and scanned in 2005, which would return patients with a mean age of approximately 12 years old. Using this approach, the mean age of subjects at the time of scan would be approximately 13 years old, regardless of the year of birth. **(B)** The search generated a total of 3,311 radiology reports, which were manually screened for exclusion criteria. The remaining 1,111 scans were imported from clinical image repositories and inspected for artifact using ChRIS software. Exclusion of 796 scans based on visual inspection yielded 315 potentially usable scans from unique individuals. These scans were manually edited by a technician who was blind to demographic information or group membership, resulting in exclusion of 23 scans for skull wrap or other non-editable artifact. This resulted in a final tally of 292 usable scans.

© 2018 Eryilmaz H et al. *JAMA Psychiatry.*

eFigure 3. Distribution of Birth Year Among MGH and PNC Cohort Individuals

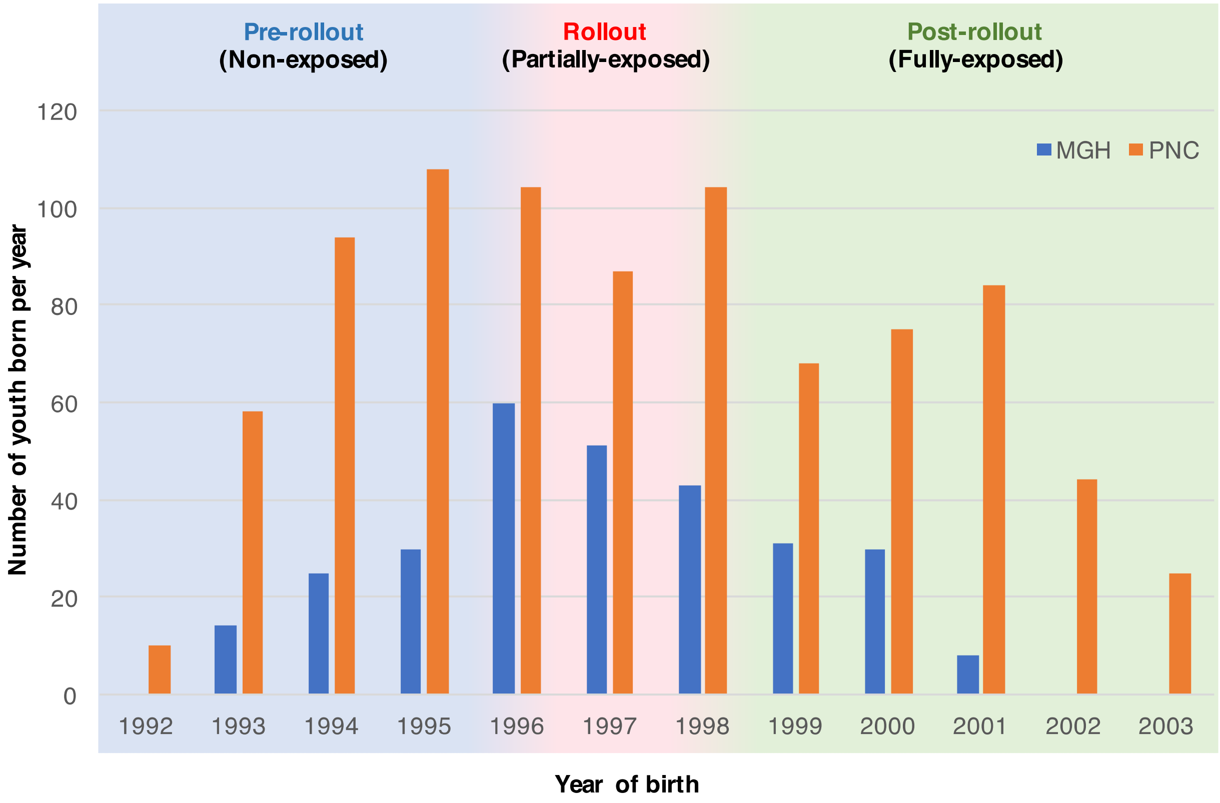

Birthdates in both cohorts were roughly centered around the time of the fortification rollout.

© 2018 Eryilmaz H et al. *JAMA Psychiatry.*

eFigure 4. Additional Clusters Demonstrating Exposure-Related Effects on Cortical Thickness in the MGH Cohort

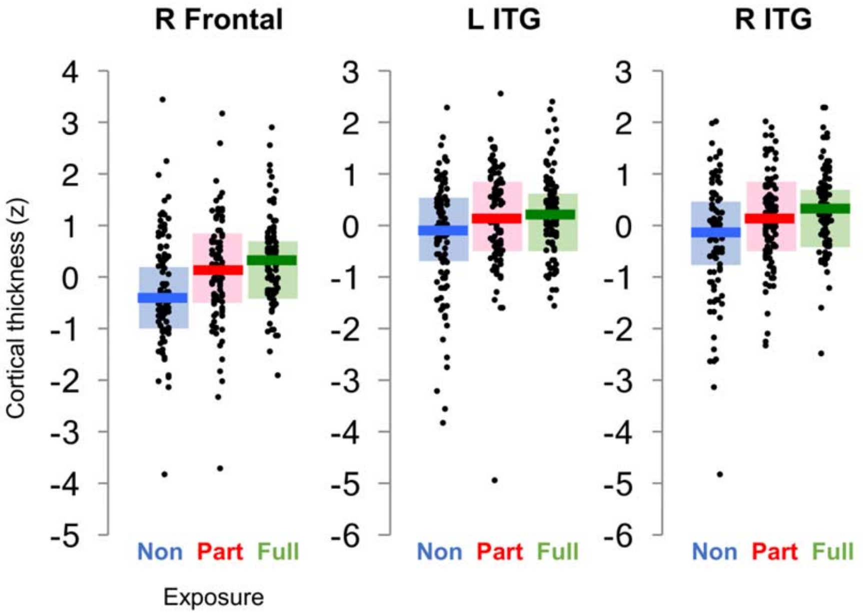

Dot plots showing cortical thickness in the right frontal and bilateral inferior temporal gyrus (ITG) as a function of exposure group (Non, nonexposed; Part, partially exposed; Full, fully exposed), suggesting intermediate effects in the partially exposed group. Bars indicate median values and shaded boxes indicate interquartile ranges. Cortical thickness values are z-transformed residuals after controlling for nuisance covariates.

© 2018 Eryilmaz H et al. *JAMA Psychiatry.*

eFigure 5. Comparison of Significant Mean Age-Centered and Intercept (Age 8)–Centered Clusters Showing Exposure-Related Effects in the MGH Cohort

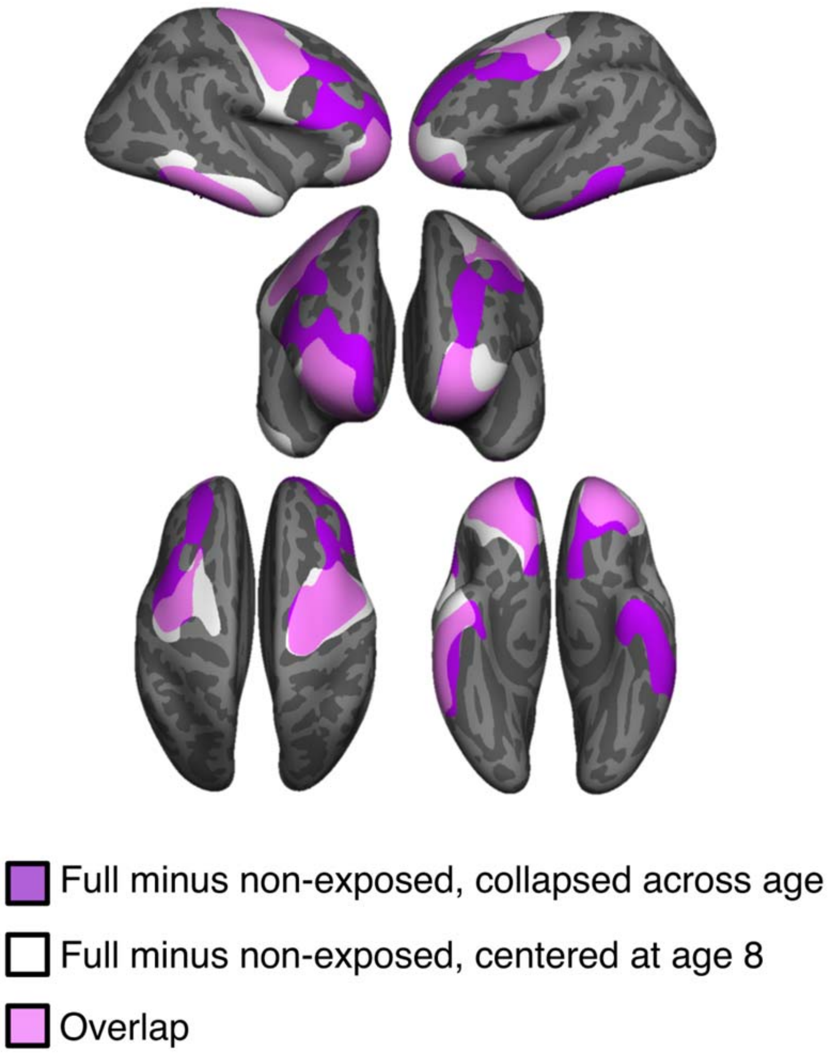

Most clusters, but not all (see, e.g., left inferior temporal gyrus), showing significant effects of exposure on cortical thickness when collapsed across age already exhibit significant differences by age 8. This pattern underscores how the strongest effects of prenatal fortification exposure occur in younger participants.

© 2018 Eryilmaz H et al. *JAMA Psychiatry.*

eFigure 6. Estimate of Thinning Delay in MGH and PNC Clusters Showing Quadratic Age-Related Thinning

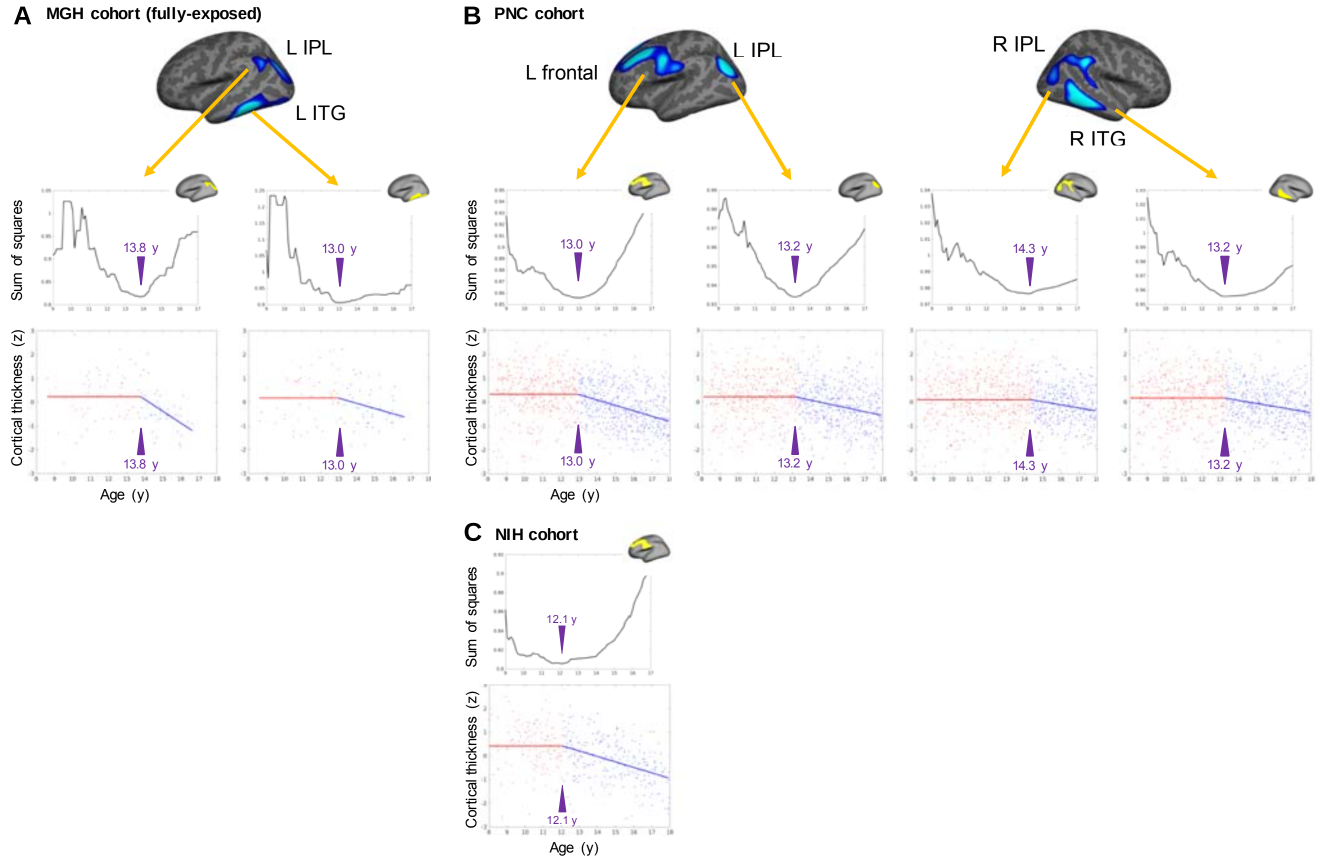

χ² = 11.87, P < .001

Estimates for the break point age, representing the age when cortical thinning begins, were developed for each MGH (A) and PNC (B) cluster showing significant quadratic thinning. "Flat-slope" models were derived for potential break points starting at age 9.0 and continuing upward by increments of 0.1 years to 17.0. For example, the function for the break point occurring at age 11.5 would consist of (1) a straight line from age=8.0 to 11.5, comprising the average thickness value for that interval, and (2) a sloped line from age 11.5 to 18.0, based on the best linear fit of all data points across that interval, further constrained so that the new sloped line was contiguous with the flat line from part (1). Mean of squares were determined for each incremental break point model, representing the goodness-of-fit of that model to the actual thickness data from participants between 8.0 and 18.0 years old. The mean of squares for each break point model were plotted as a function of break point age; the nadir of the resulting curve reflected the break point age that optimally fit the data. Below, the original cortical thickness versus age data are plotted, indicating at what age the optimal break point occurred. Red data points comprise the "flat" part, and blue data points comprise the "slope" part. Accordingly, the data suggest that cortical thinning among fortification-exposed individuals began between ages 13.0 and 14.3, depending on region. **(C)** Among the six regions showing quadratic thinning in the MGH and PNC cohorts, five showed no evidence of quadratic thinning in the nonexposed NIH cohort. As such, thinning had already begun by age 8 among NIH participants. However, one region, the left frontal cortex cluster derived in the PNC cohort, did exhibit significant quadratic thinning within the NIH cohort – suggesting that for that region, quadratic thinning was present before the onset of fortification. However, break point analysis for the NIH data in this cluster indicated an earlier onset of thinning (age 12.1) than that observed within the same cluster in the PNC data (age 13.0). Chi-square analysis of the NIH data comparing distributions of data before versus after the two break points indicated a significant difference in thinning onset.

© 2018 Eryilmaz H et al. *JAMA Psychiatry.*

eFigure 7. Additional Clusters Demonstrating Quadratic Age-Related Thinning in the PNC Cohort

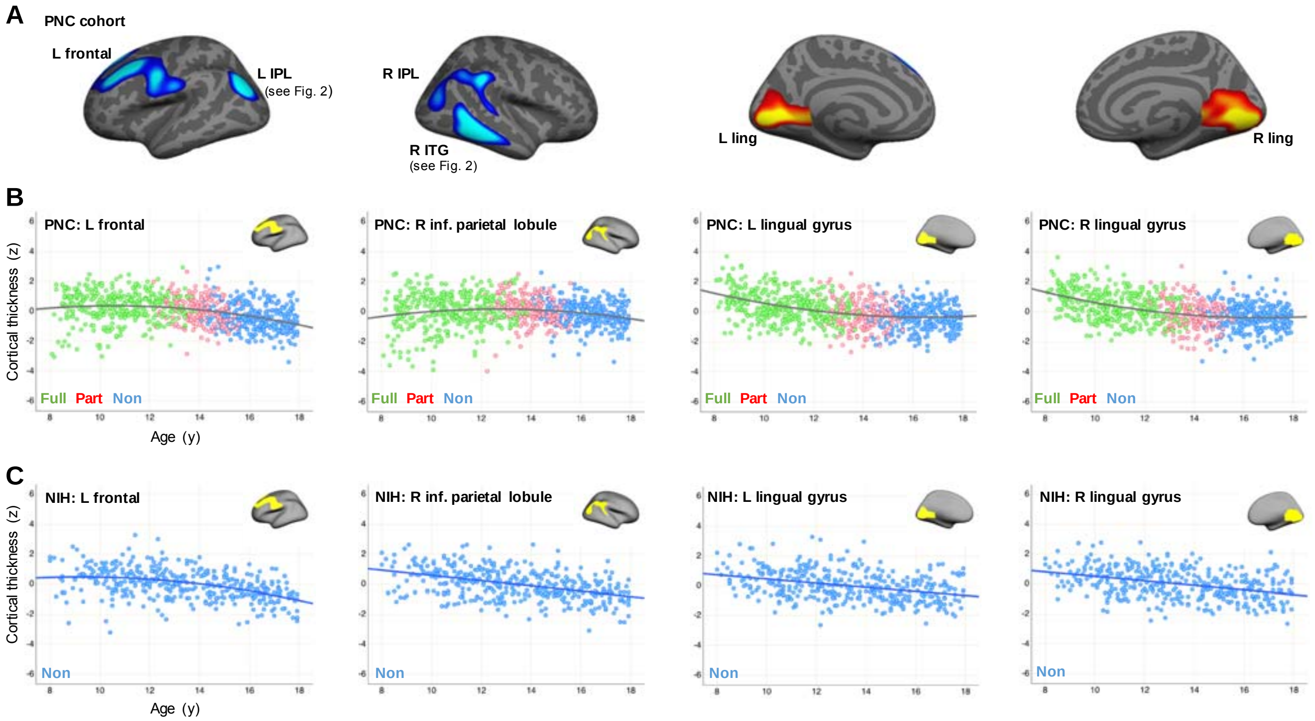

(A) Surface-wide maps of age-squared effects on cortical thickness in the PNC cohort (N=861) indicate quadratic age-related thinning, driven largely by individuals who were fully exposed to fortification during gestation. Consistent with the MGH cohort, delayed thinning occurred in left frontal, bilateral inferior parietal lobule (IPL, extending to supramarginal gyrus on the right side), and right inferior temporal gyrus (ITG). Unlike the MGH cohort, accelerated thinning was seen in bilateral lingual gyrus. **(B)** Age-thickness scatterplots depicting subject-level data from panel A. **(C)** Within in all but one of these clusters, only linear thinning was seen in the nonexposed NIH cohort (N=383 scans), the exception being the left frontal cluster (see also eFigure 6). Images in panel A are masked to show only clusters that survive correction for multiple comparisons (p<.05, clusterwise). Cortical thickness values in scatterplots represent z-transformed residuals after controlling for nuisance covariates.

© 2018 Eryilmaz H et al. *JAMA Psychiatry.*

eTable 1. Radiologic Exclusion Criteria for MGH Cohort

Exclusion criterion	N scans
Multiple exclusion criteria	291
Surgery (craniotomy, craniectomy, lobectomy, resection)	227
Tumor (astrocytoma, glioma, other neoplasms)	197
Tuberous sclerosis	186
Artifact (metal, dental hardware, susceptibility artifact)	186
Artifact (motion)	168
Multiple or large FLAIR or T2 signal hyperintensities	94
Unspecified lesion	94
Other anatomical abnormalities	89
Last known address outside of United States or Puerto Rico	85
Early scan termination, incomplete scan, or non-brain MRI	77
Cyst demonstrating mass effect or >1 cm diameter	76
Hemorrhage or encephalomalacia	73
Patient deceased after scan	48
Ventriculostomy and/or shunt	35
Multiple sclerosis	35
Focal cortical dysplasia	29
Post-operative follow-up	27
Chiari malformation	26
Post-radiation to head	24
Leukodystrophy	20
Developmental venous anomaly	15
Neurofibromatosis	15
Gray matter heterotopia	14
Infarction	13
Volume loss	12
Traumatic injury	11
Hydrocephalus or ventriculomegaly	9
Mesial temporal sclerosis	7
Outside MRI scan read at MGH	6
Polymicrogyria	4
Neurocysticercosis	3
Neurometabolic disorder	2
Gliosis	2

© 2018 Eryilmaz H et al. *JAMA Psychiatry.*

eTable 2. Comparison of Included and Excluded Individuals in MGH Cohort

	Used in analysis	Screened out (image quality)	Screened out (pathology)
**Age (mean ± SD)**	13.3 ± 2.3	13.5 ± 2.2	13.2 ± 2.1
**Sex (% female)**	47.3	51.3	46.7
**Race (%)**			
African	5.1	5.4	4.8
Asian	3.4	3.0	3.6
Caucasian	72.9	73.1	75.4
Hispanic	9.2	7.2	6.7
Not recorded	4.8	5.0	4.5
Other	4.5	6.4	5.0

© 2018 Eryilmaz H et al. *JAMA Psychiatry.*

eTable 3. Clusters Demonstrating Significantly Increased Cortical Thickness in Fully Exposed Versus Nonexposed Youths in MGH Cohort
(A) Significant clusters when collapsed across age. (B) Significant clusters occurring at age 8. NB: no clusters demonstrated increased cortical thickness in nonexposed versus fully exposed youths.

A

Hemi	Region	Cluster size (mm^2)	Cluster -wise *p*-value	Peak MNI[a] x, y, z coordinates	Peak vertex *p*-value	Group difference (mm, 95% C.I.)	Thickness range (mm)	% Diff.
Left	Inferior temporal gyrus	2,666	0.03	-54, -32, -28	0.00003	0.16 (0.09-0.24)	1.61	9.9
	Frontal (peak: precentral)	7,219	0.0001	-49, -2, 47	0.00009	0.12 (0.07-0.17)	1.19	10.1
Right	Inferior temporal gyrus	2,672	0.03	58, -24, -30	0.00005	0.16 (0.09-0.23)	1.52	10.5
	Frontal (peak: precentral)	11,810	0.0001	44, 1, 46	0.00009	0.10 (0.06-0.15)	1.86	11.6

[a]MNI, Montreal Neurological Institute

B

Hemi	Region	Cluster size (mm^2)	Cluster -wise *p*-value	Peak MNI[a] x, y, z coordinates	Peak vertex *p*-value
Left	Frontal (peak: pars orbitalis)	3,611	0.003	-42, -43, -9	0.00002
	Frontal (peak: precentral)	2,581	0.030	-34, -18, 38	0.0003
Right	Frontal (peak: pars orbitalis)	4,071	0.001	33, 45, -11	0.00009
	Frontal (peak: precentral)	5,487	0.0001	55, -4, 36	0.0001
	Inferior temporal gyrus	3,671	0.003	48, -7, -36	0.001

[a]MNI, Montreal Neurological Institute

© 2018 Eryilmaz H et al. *JAMA Psychiatry.*

eTable 4. Effects of Scanner Strength and Manufacturer Differences on Group Cortical Thickness Differences in MGH Cohort
(A) Main effect of group (fully vs. nonexposed) on cortical thickness, covaried for nuisance covariates (age, age^2, sex, and total brain volume), as well as region-specific effects. (B) As in part A, now including scanner field (1.5 vs. 3T) and scanner field x group interaction terms. (C) As in part A, now including manufacturer (GE vs. Siemens) and manufacturer x group interaction terms.

A

	Mean thickness (mm ± S.E.)		F	*p*-value
	Nonexp.	Full exp.		
Main effect of group			11.17	3.8×10^{-8}
Right inferior temporal gyrus	3.13 ± 0.02	3.32 ± 0.02	39.82	1.9×10^{-9}
Right frontal	2.71 ± 0.02	2.77 ± 0.02	6.38	0.012
Left inferior temporal gyrus	3.09 ± 0.02	3.28 ± 0.02	34.32	2.0×10^{-8}
Left frontal	2.77 ± 0.01	2.83 ± 0.01	9.98	0.002

B

	Mean thickness (mm ± S.E.)		F	*p*-value
	Nonexp.	Full exp.		
Main effect of group			7.03	0.00003
Right inferior temporal gyrus	3.14 ± 0.03	3.30 ± 0.02	20.51	0.00001
Right frontal	2.68 ± 0.02	2.80 ± 0.02	21.04	0.00008
Left inferior temporal gyrus	3.11 ± 0.03	3.25 ± 0.02	15.38	0.0001
Left frontal	2.75 ± 0.02	2.85 ± 0.02	19.71	0.00002
Main effect of scanner field strength			12.87	2.9×10^{-9}
Group x field strength interaction			0.46	0.76

C

	Mean thickness (mm ± S.E.)		F	*p*-value
	Nonexp.	Full exp.		
Main effect of group			6.48	0.00007
Right inferior temporal gyrus	3.16 ± 0.02	3.30 ± 0.02	19.18	0.00002
Right frontal	2.70 ± 0.02	2.80 ± 0.02	17.34	0.00005
Left inferior temporal gyrus	3.12 ± 0.02	3.26 ± 0.03	15.81	0.00001
Left frontal	2.77 ± 0.02	2.86 ± 0.02	18.00	0.00004
Main effect of scanner manufacturer			13.46	1.2×10^{-9}
Group x manufacturer interaction			1.16	0.33

© 2018 Eryilmaz H et al. *JAMA Psychiatry.*

eTable 5. Clusters Demonstrating Significantly Different Quadratic Age-Related Thinning in Fully Exposed Versus Nonexposed Youths in MGH Cohort

Hemi	Region	Direction (full relative to non-exp)	Cluster size (mm^2)	Clusterwise *p*-value	Peak MNI[a] x, y, z coordinates	Peak vertex *p*-value	β: age^2 x group
**Left**	Inferior temporal gyrus	Delayed	3,829	0.002	-57, -46, -19	0.00001	-13.9
	Inferior parietal lobule	Delayed	3,724	0.002	-34, -82, 30	0.0006	-11.1

[a]MNI, Montreal Neurological Institute

© 2018 Eryilmaz H et al. *JAMA Psychiatry.*

eTable 6. Clusters Demonstrating Quadratic Age-Related Thinning in PNC Cohort

Hemi	Region	Direction (full relative to nonexp)	Cluster size (mm^2)	Clusterwise *p*-value	Peak MNI[a] x, y, z coordinates	Peak vertex *p*-value	β: age^2
**Left**	Inferior parietal lobule	Delayed	1,704	0.006	-42, -74, 31	0.0000003	-1.59
	Frontal (peak: caudal middle frontal)	Delayed	6,390	0.0001	-36, 21, 48	0.00001	-1.70
	Lingual gyrus	Accelerated	3,544	0.002	-7, -85, 0	0.00002	1.53
**Right**	Inferior-middle temporal gyrus	Delayed	2,738	0.02	58, -44, -13	0.00004	-1.73
	Lingual gyrus	Accelerated	5,066	0.0001	6, -86, -8	0.00005	1.55
	Inferior parietal-supramarginal gyrus	Delayed	3,444	0.004	58, -40, 34	0.0001	-1.61

[a]MNI, Montreal Neurological Institute

© 2018 Eryilmaz H et al. *JAMA Psychiatry.*

eTable 7. Tests for Quadratic Age-Related Thinning in NIH Cohort, Within Clusters Demonstrating Significant Quadratic Thinning in MGH or PNC Cohorts

Hemisphere	Region	F: age^2	*p*-value
**Regions with quadratic (delayed) thinning in MGH cohort**			
**Left**	Inferior temporal gyrus	1.48	0.23
	Inferior parietal lobule	0.01	0.92
**Regions with quadratic (delayed) thinning in PNC cohort**			
**Left**	Inferior parietal lobule	1.25	0.27
	Frontal (peak: caudal middle frontal)	8.87	0.003
**Right**	Inferior-middle temporal gyrus	2.68	0.10
	Inferior parietal-supramarginal gyrus	0.72	0.40

© 2018 Eryilmaz H et al. *JAMA Psychiatry.*

eTable 8. Quadratic Age-Related Thinning in PNC Cohort Before and After Exclusion of Youths With Psychotic Symptoms

Hemisphere	Region	All subjects (N=861)		PS[a]/PL[b] subjects excluded (N=541)	
		β: age^2	ROI[c] *p*-value	β: age^2	ROI *p*-value
**Left**	Inferior parietal lobule	-1.59	0.000003	-1.48	0.0008
	Frontal (peak: caudal middle frontal)	-1.70	4.9×10^{-7}	-1.82	0.00004
	Lingual gyrus	1.53	0.000002	1.71	0.00006
**Right**	Inferior-middle temporal gyrus	-1.73	0.000001	-1.91	0.00005
	Lingual gyrus	1.55	9.6×10^{-7}	1.87	0.000007
	Inferior parietal lobule-supramarginal gyrus	-1.61	0.000004	-1.77	0.0001

[a]PS, Psychosis Spectrum; [b]PL, Psychosis Low; [c]ROI, Region-of-interest statistics. The ROI was defined based on surface-wide analysis of the N=861 cohort.

© 2018 Eryilmaz H et al. *JAMA Psychiatry.*

eReferences

1. Satterthwaite TD, Elliott MA, Ruparel K, et al. Neuroimaging of the Philadelphia neurodevelopmental cohort. *Neuroimage.* 2014;86:544-553.

2. Vandekar SN, Shinohara RT, Raznahan A, et al. Topologically dissociable patterns of development of the human cerebral cortex. *J Neurosci.* 2015;35(2):599-609.

3. Merikangas KR, Calkins ME, Burstein M, et al. Comorbidity of physical and mental disorders in the neurodevelopmental genomics cohort study. *Pediatrics.* 2015;135(4):e927-938.

4. Waber DP, De Moor C, Forbes PW, et al. The NIH MRI study of normal brain development: performance of a population based sample of healthy children aged 6 to 18 years on a neuropsychological battery. *J Int Neuropsychol Soc.* 2007;13(5):729-746.

5. Evans AC, Brain Development Cooperative G. The NIH MRI study of normal brain development. *Neuroimage.* 2006;30(1):184-202.

6. Ducharme S, Albaugh MD, Nguyen TV, et al. Trajectories of cortical thickness maturation in normal brain development--The importance of quality control procedures. *Neuroimage.* 2016;125:267-279.

7. Pienaar R, Rannou N, Bernal J, Hahn D, Grant PE. ChRIS--A web-based neuroimaging and informatics system for collecting, organizing, processing, visualizing and sharing of medical data. *Conf Proc IEEE Eng Med Biol Soc.* 2015;2015:206-209.

8. Dale AM, Fischl B, Sereno MI. Cortical surface-based analysis. I. Segmentation and surface reconstruction. *Neuroimage.* 1999;9(2):179-194.

9. Fischl B, Sereno MI, Dale AM. Cortical surface-based analysis. II: Inflation, flattening, and a surface-based coordinate system. *Neuroimage.* 1999;9(2):195-207.

10. Fischl B, Dale AM. Measuring the thickness of the human cerebral cortex from magnetic resonance images. *Proc Natl Acad Sci U S A.* 2000;97(20):11050-11055.

11. Desikan RS, Segonne F, Fischl B, et al. An automated labeling system for subdividing the human cerebral cortex on MRI scans into gyral based regions of interest. *Neuroimage.* 2006;31(3):968-980.

12. Rosas HD, Liu AK, Hersch S, et al. Regional and progressive thinning of the cortical ribbon in Huntington's disease. *Neurology.* 2002;58(5):695-701.

13. Kuperberg GR, Broome MR, McGuire PK, et al. Regionally localized thinning of the cerebral cortex in schizophrenia. *Arch Gen Psychiatry.* 2003;60(9):878-888.

14. Han X, Jovicich J, Salat D, et al. Reliability of MRI-derived measurements of human cerebral cortical thickness: the effects of field strength, scanner upgrade and manufacturer. *Neuroimage.* 2006;32(1):180-194.

15. Reuter M, Schmansky NJ, Rosas HD, Fischl B. Within-subject template estimation for unbiased longitudinal image analysis. *Neuroimage.* 2012;61(4):1402-1418.

16. Govindarajan KA, Freeman L, Cai C, Rahbar MH, Narayana PA. Effect of intrinsic and extrinsic factors on global and regional cortical thickness. *PLoS One.* 2014;9(5):e96429.

17. Calkins ME, Moore TM, Merikangas KR, et al. The psychosis spectrum in a young U.S. community sample: findings from the Philadelphia Neurodevelopmental Cohort. *World Psychiatry.* 2014;13(3):296-305.

© 2018 Eryilmaz H et al. *JAMA Psychiatry.*

18. Satterthwaite TD, Wolf DH, Calkins ME, et al. Structural Brain Abnormalities in Youth With Psychosis Spectrum Symptoms. *JAMA Psychiatry.* 2016;73(5):515-524.

19. Gur RC, Calkins ME, Satterthwaite TD, et al. Neurocognitive growth charting in psychosis spectrum youths. *JAMA Psychiatry.* 2014;71(4):366-374.

20. Kobayashi H, Nemoto T, Koshikawa H, et al. A self-reported instrument for prodromal symptoms of psychosis: testing the clinical validity of the PRIME Screen-Revised (PS-R) in a Japanese population. *Schizophr Res.* 2008;106(2-3):356-362.

21. Kaufman J, Birmaher B, Brent D, et al. Schedule for Affective Disorders and Schizophrenia for School-Age Children-Present and Lifetime Version (K-SADS-PL): initial reliability and validity data. *J Am Acad Child Adolesc Psychiatry.* 1997;36(7):980-988.

22. Miller TJ, McGlashan TH, Rosen JL, et al. Prodromal assessment with the structured interview for prodromal syndromes and the scale of prodromal symptoms: predictive validity, interrater reliability, and training to reliability. *Schizophr Bull.* 2003;29(4):703-715.

23. Kelleher I, Connor D, Clarke MC, Devlin N, Harley M, Cannon M. Prevalence of psychotic symptoms in childhood and adolescence: a systematic review and meta-analysis of population-based studies. *Psychol Med.* 2012;42(9):1857-1863.

© 2018 Eryilmaz H et al. *JAMA Psychiatry.*
